# Spatial diversity patterns of *Pristimantis* frogs in the Tropical Andes

**DOI:** 10.1002/ece3.1968

**Published:** 2016-02-19

**Authors:** Fabio Leonardo Meza‐Joya, Mauricio Torres

**Affiliations:** ^1^ Grupo de Estudios en BiodiversidadFacultad de Ciencias, Escuela de BiologíaUniversidad Industrial de SantanderBucaramanga680002Colombia; ^2^ Fundación IguaqueBucaramanga680002Colombia

**Keywords:** Diversity gradient, mid‐domain effect, spatial hypothesis, species richness, species–area relationships, topographic heterogeneity

## Abstract

Although biodiversity gradients have been widely documented, the factors governing broad‐scale patterns in species richness are still a source of intense debate and interest in ecology, evolution, and conservation biology. Here, we tested whether spatial hypotheses (species–area effect, topographic heterogeneity, mid‐domain null model, and latitudinal effect) explain the pattern of diversity observed along the altitudinal gradient of Andean rain frogs of the genus *Pristimantis*. We compiled a gamma‐diversity database of 378 species of *Pristimantis* from the tropical Andes, specifically from Colombia to Bolivia, using records collected above 500 m.a.s.l. Analyses were performed at three spatial levels: Tropical Andes as a whole, split in its two main domains (Northern and Central Andes), and split in its 11 main mountain ranges. Species richness, area, and topographic heterogeneity were calculated for each 500‐m‐width elevational band. Spatial hypotheses were tested using linear regression models. We examined the fit of the observed diversity to the mid‐domain hypothesis using randomizations. The species richness of *Pristimantis* showed a hump‐shaped pattern across most of the altitudinal gradients of the Tropical Andes. There was high variability in the relationship between area and species richness along the Tropical Andes. Correcting for area effects had little impact in the shape of the empirical pattern of biodiversity curves. Mid‐domain models produced similar gradients in species richness relative to empirical gradients, but the fit varied among mountain ranges. The effect of topographic heterogeneity on species richness varied among mountain ranges. There was a significant negative relationship between latitude and species richness. Our findings suggest that spatial processes partially explain the richness patterns of *Pristimantis* frogs along the Tropical Andes. Explaining the current patterns of biodiversity in this hot spot may require further studies on other possible underlying mechanisms (e.g., historical, biotic, or climatic hypotheses) to elucidate the factors that limit the ranges of species along this elevational gradient.

## Introduction

The unequal distribution of biodiversity on the world is a crucial and still unresolved issue (Kennedy and Norman 2005) that has captivated biogeographers and ecologists for centuries (Lomolino 2001; McCain and Grytnes 2010; Hu et al. 2011). Although gradients of species diversity have been widely documented, the mechanisms responsible for differences in geographic and taxonomic distribution of biological diversity are still a source of intense debate (Pianka 1966; Lomolino 2001; Rahbek 2005; Stevens et al. 2013; Graham et al. 2014). One of the main gradients of diversity observed in nature is that formed by elevation. The altitudinal patterns of diversity have been studied only recently for many groups of plants and animals (Rahbek 1995; Heaney 2001; Brehm et al. 2003; McCain 2005, 2009, 2010; Pyrcz et al. 2013; among others), refining our understanding of elevational gradients. For example, early naturalists (von Humboldt, Darwin, and Wallace) proposed that a decreasing pattern was typical along tropical altitudinal gradients (Lomolino 2001), but other patterns of diversity have been observed over recent years (McCain and Grytnes 2010).

Despite the growing efforts to describe global trends of biodiversity (Jetz and Rahbek 2002; Rahbek 2005; McCain 2009, 2010; Jetz and Fine 2012), there is still a need for the analysis of more altitudinal patterns, especially in the tropical regions where biodiversity is high but poorly sampled. Recent studies have found four common elevation diversity patterns around the world (Rahbek 1995; Lomolino 2001; McCain 2009; McCain and Grytnes 2010). In the decreasing pattern, the number of species decreases monotonically from low‐to‐high elevations. In the low plateau pattern, richness shows a plateau at the lower portion of the gradient and then the species numbers decline with increasing elevation. In the low plateau with a mid‐elevational peak, the highest richness forms a peak near of the low elevation limit of the range. In the mid‐elevation peak (i.e., hump‐shaped or unimodal pattern), the highest richness is found at intermediate elevations, with the species number decreasing toward the base and top of the mountains. Despite the megadiversity of the Andean biota, currently the diversity patterns of only a handful of Andean taxa have been investigated (birds: Rahbek 1997; Kattan and Franco 2004; mammals: McCain 2007a; frogs: Hutter et al. 2013; ferns: Salazar et al. 2015; among others).

The explanations for observed altitudinal patterns can be classified as climatic, evolutionary, biotic, and spatial (Wiens et al. 2007; McCain and Grytnes 2010; Acharya et al. 2011). Climate has been evoked as a strong driver of species richness gradients in many taxonomic groups, with temperature, precipitation, and productivity as the most commonly studied climatic variables (e.g., Hawkins et al. 2003; Rodríguez et al. 2005; McCain 2010). Evolutionary history (referring to speciation rates, extinction rates, clade age, and phylogenetic niche conservatism) explains some elevational diversity patterns (e.g., Smith et al. 2007; Wiens et al. 2007; Hutter et al. 2013). Biotic processes and biological interactions (such as ecotone effects, source‐sink dynamics, habitat heterogeneity, habitat complexity, competition, and mutualism) are also related to patterns in species richness (e.g., Terborgh 1977; Lomolino 2001; McCain and Grytnes 2010). Spatial hypotheses, including SAR, mid‐domain effect (MDE), and spatial environmental heterogeneity (SEH), explain some elevation species richness patterns for many taxonomic groups (e.g., Rahbek 1995, 2005; Fleishman and Mac Nally 2002; Fu et al. 2006; McCain 2007a; Chettri et al. 2010; Hu et al. 2011; Stevens et al. 2013; Stein et al. 2014), but these type of analyses are rare for Neotropical biota, which is recognized as the most diverse of the world.

Here, we present the first of a series of studies intended to investigate the patterns of species richness of the genus *Pristimantis* along elevational gradients in the Tropical Andes. As a first step, we tested whether the diversity patterns can be explained by spatial hypothesis, while accounting for area effects. Three major hypotheses SARs, MDE, and SEH have been proposed to explain spatial patterns of diversity.

Species–area relationships predict a positive relationship between species richness and survey area based on the assumption that more area can bear more species (Rosenzweig 1995). On mountains, SAR may explain a decreasing richness pattern of diversity when the lower elevations have more land than high elevations (Rahbek 1997; McCain 2007a). The same occurs in gradients with more land area at mid‐elevations, producing a pattern with a mid‐peak of high richness (McCain and Grytnes 2010). However, the support for this hypothesis is contradictory because the correlation between area and diversity varies from positive to null to even negative (Sanders 2002; McCain 2007a, 2009, 2010).

The mid‐domain effect is a mid‐elevation peak of biodiversity based in the stochastic distribution produced by randomly shuffling ranges of distribution within geographic constraints (Colwell and Hurtt 1994; Colwell and Lees 2000; Colwell et al. 2004). The constraints may be latitudinal (i.e., latitudes are circumscribed between the poles) or terrestrial (i.e., land is restricted between oceans and elevation of mountain peaks). The conceptual base of MDE has been a hot topic and much controversy has surrounded the assumptions of this model (Koleff and Gaston 2001; Hawkins and Diniz‐Filho 2002; Zapata et al. 2003, 2005). Despite many studies supporting the mid‐domain model predictions, others have found little support, suggesting that this model is not a general explanation for diversity patterns (Hawkins and Diniz‐Filho 2002; Kerr et al. 2006; Dunn et al. 2007).

Spatial environmental heterogeneity may be another determinant of species diversity. Heterogeneous environments can harbor more species, enhance species persistence, and promote adaptive radiations because they can have a rich array of suitable conditions, such as topographic complexity, niche availability, resources, shelter, and refuges (Rosenzweig 1995; Thuiller et al. 2006; Antonelli and Sanmartín 2011; Allouche et al. 2012; Fjeldså et al. 2012; Stein et al. 2014). Although environmental heterogeneity has been recognized as a fundamental driver of species richness, evidence supporting this model varies from significant to nonsignificant or even negative effects (e.g., Fleishman and Mac Nally 2002; Tews et al. 2004; Hortal et al. 2009; Tamme et al. 2010; Gazol et al. 2013; Laanisto et al. 2013; Stein et al. 2014).

Because the Tropical Andes have a wide latitudinal range, we considered latitude as another key spatial factor for the distribution of diversity. Latitudinal gradients are perhaps the most noticeable and best‐studied patterns in ecology (Gaston 2000; Sanders and Rahbek 2012; Salazar et al. 2015). With few exceptions (Clarke and Lidgard 2000), it has been found across taxa that species richness increases with decreasing latitude. However, the causes determining these patterns are still being discussed (Pianka 1966; Rohde 1992; Rosenzweig 1995; Willig et al. 2003; Pyrcz et al. 2013; Salazar et al. 2015).

**Figure 1:**
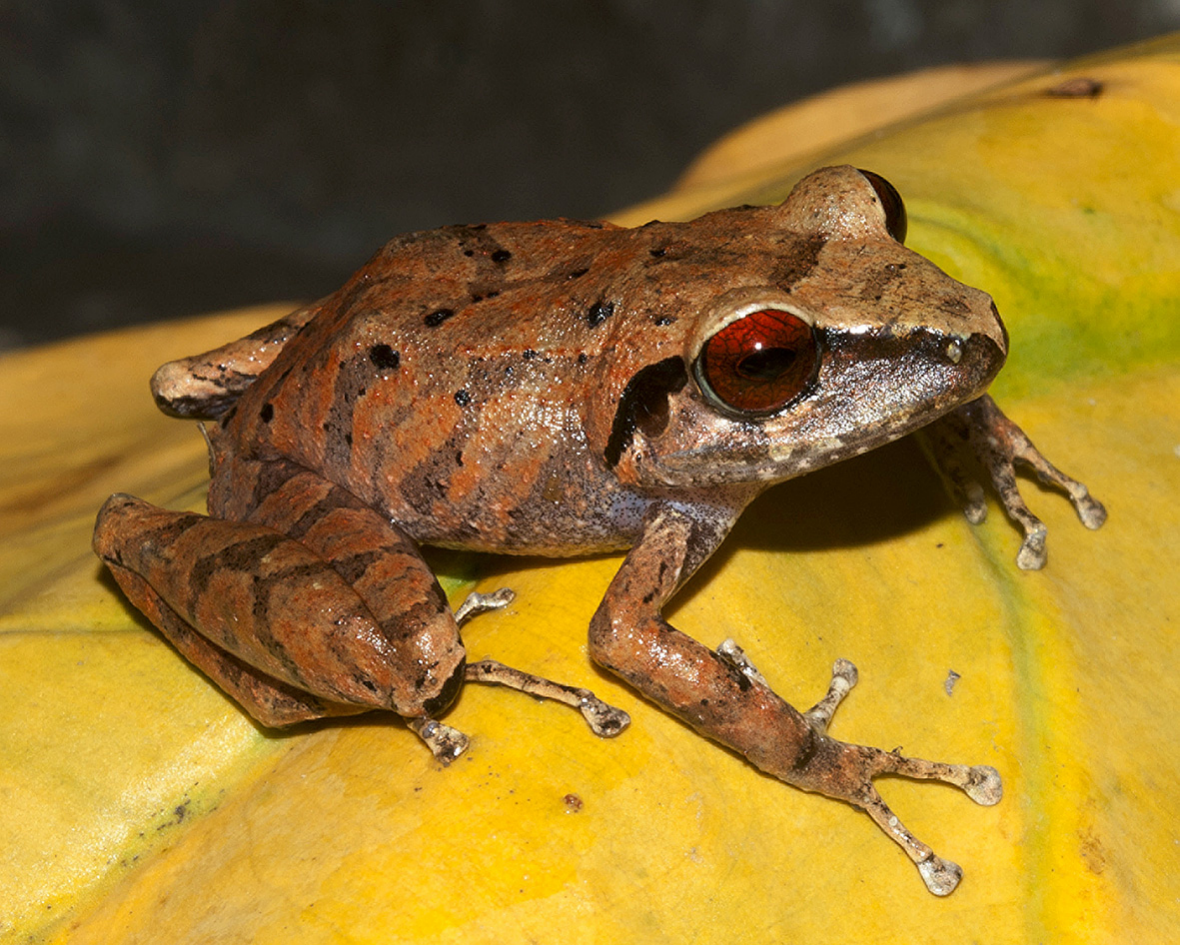
Example of one of the species included in this study: *Pristimantis bacchus*, an endemic rain frog from Tropical Andes in Colombia.

Neotropical direct‐developing *Pristimantis* frogs (Caugastoridae sensu Padial et al. 2014; Fig. 1) form an excellent group for a large‐scale study of diversity and distribution. These frogs comprise a major group of amphibians with more than 470 species (Padial et al. 2014; AmphibiaWeb, 2015). Most species of *Pristimantis* occur in moist and forested habitat of the Tropical Andes of Colombia, Ecuador, and Peru (Lynch and Duellman 1997; Pinto‐Sánchez et al. 2012). The elevational range of the genus is broad, from species living at sea level to some occurring above 4500 m (Heinicke et al. 2007). There are a number of studies on the taxonomy, phylogenetics, and biogeography of *Pristimantis* frogs (García‐R et al. 2012; Pinto‐Sánchez et al. 2012; Padial et al. 2014; among others). However, the large‐scale distribution patterns of these frogs are not well understood.

To investigate the patterns of species richness of *Pristimantis* frogs along elevational and latitudinal gradients of the Tropical Andes, we used published data on elevation ranges. First, we described the elevational richness patterns of *Pristimantis* frogs in the Andes Mountains. Then, we assessed how much of the observed elevation patterns of diversity could be explained by area. Third, we tested whether MDE can explain the empirical patterns along these altitudinal gradients, while accounting for any SAR. Fourth, we examined the influence of spatial topographic heterogeneity (as a surrogate of SEH) and latitude on the observed diversity patterns. Our results are important to increase our current comprehension of the mechanisms promoting and maintaining the amphibian fauna in the Tropical Andes and to identify the most plausible schemes for conservation of the Andean biodiversity.

## Materials and Methods

### Study region

**Figure 2:**
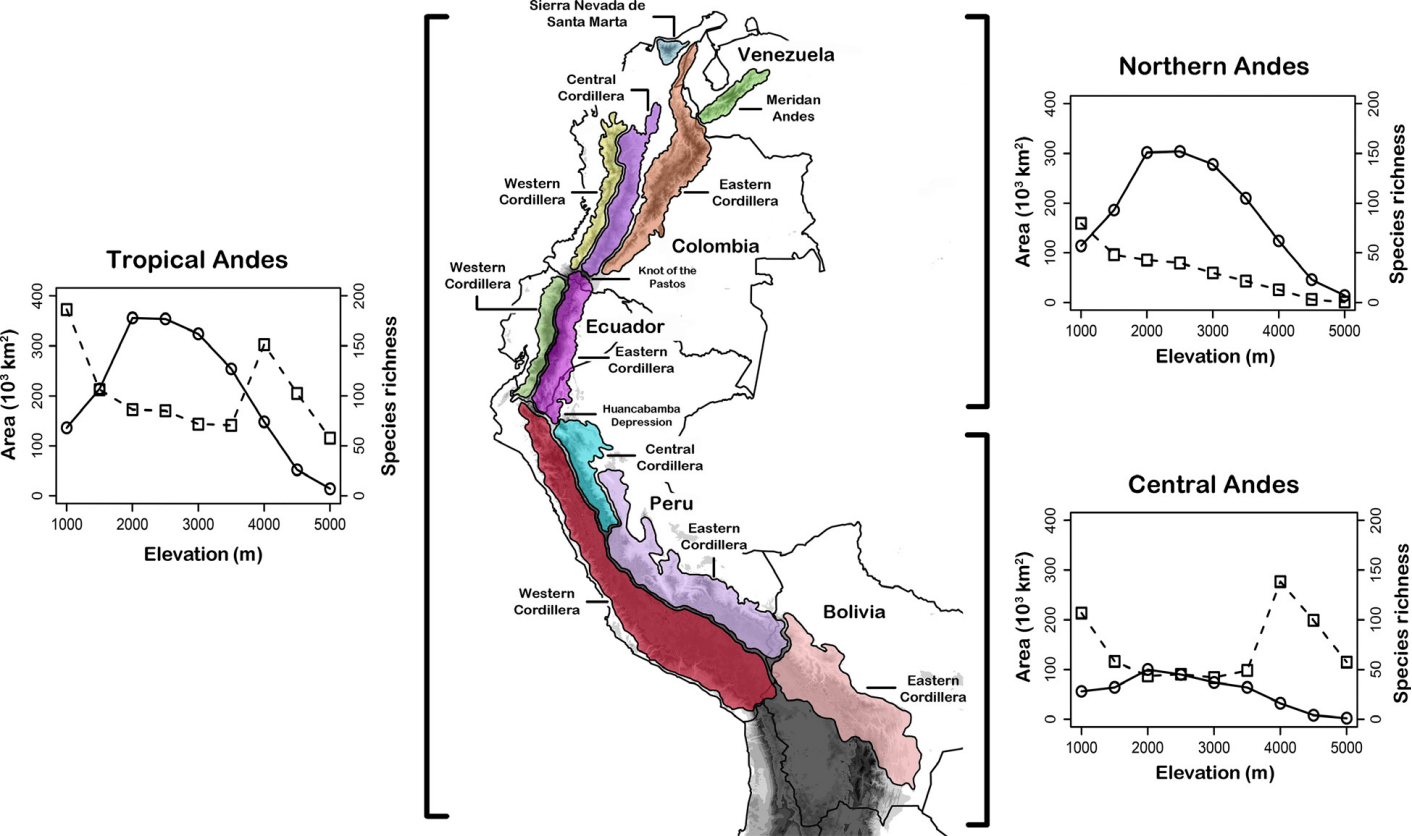
Map of South America indicating the Tropical Andes (dark relief) with the main domains and mountain ranges (or cordilleras) where the genus *Pristimantis* occurs. Lateral figures show the patterns of area (open squares and dotted lines) and species richness (solid circles and solid lines) for the Tropical Andes, Northern Andes, and Central Andes.

The Tropical Andes extend along the western coast of South America, from Venezuela to northern Chile and Argentina, including extensive areas of Colombia, Ecuador, Peru, and Bolivia (Myers et al. 2000). This region includes many of the Earth's life zones and is considered a biodiversity hot spot due to high species richness and endemism (Myers et al. 2000; Young 2011). Although the topography of the Tropical Andes is a complex array of mountain ranges and basins, the region is commonly divided in two domains, Northern and Central Andes (Gregory‐Wodzicki 2000). The Northern Andes comprises of seven mountain ranges north of the Huancabamba depression, whereas the Central Andes includes the largest areas of Andean highlands and comprises of six main cordilleras located south of that depression (Fig. 2). Because the western cordillera of the Bolivian Andes does not harbor any species of *Pristimantis* frogs, it was excluded from our study. For a detailed description of these mountain ranges, see Duellman (1979), Duellman and Pramuk (1999), and Duellman and Lehr (2009). Here, we consider 500 m.a.s.l., a commonly used value (Anderson et al. 2011) as the lower elevation limit of the tropical Andes.

### Species richness pattern

To estimate altitudinal richness patterns, we compiled a gamma‐diversity database of the Andean species of *Pristimantis* frogs (see Appendix S1 in Supporting information, Table S1). We followed the taxonomic proposal of Padial et al. (2014) to define the genus *Pristimantis*. Elevational data were obtained in November 2013, primarily from the Amphibian Species of the World database (Frost 2013) and the Global Amphibian Assessment initiative (http://www.iucnredlist.org). These data were filtered based on original species descriptions, range extension notes, and well‐supported observations (e.g., records of global networks of biodiversity and online museum catalogues). The occurrence records were verified by experts on the *Pristimantis* of each country included in the analysis (see “Acknowledgments”). The elevational range of each species was standardized by interpolation: a method that assumes continuous ranges between the minimum and maximum altitudinal records.

Species richness was defined as the number of species occurring in each of nine 500‐m‐wide altitudinal bands, following standard practice for elevational species richness studies (e.g., Rahbek 1997; Smith et al. 2007; Kozak and Wiens 2010; Hutter et al. 2013). We tested other bandwidths that were either wider (i.e., 1000 m) or narrower (i.e., 250 m) than 500 m. The analyses with these bands are not shown because wider bands were too few for statistical analysis and narrower bands were qualitatively the same results than those using 500‐m‐wide bands. For posterior analysis, we used only data from 500‐m‐wide altitudinal bands, to allow easier comparison with the results from other studies.

### Species–area relationship

Spurious elevational diversity patterns may be due to differences in postsampling treatment of data (Rahbek 1995; McCoy 2002; Nogués‐Bravo et al. 2008; McCain and Grytnes 2010). Rahbek (1995) showed that the pattern where diversity decreases with altitude is in some cases the outcome of nonstandardized samples with respect to area, because elevation bands usually vary in area. Once samples are standardized, a decreasing trend sometimes reveals itself as a hump‐shaped pattern. To examine the influence of area on the observed gamma‐diversity curves, we calculated the area of each 500‐m elevational band to Tropical Andes, each Andean domain, and each mountain range. The area was calculated using a global digital elevation model (GTOPO30) in Quantum GIS software (QGIS Development Team, 2013). The general relationship between the species richness and the size of the area was examined with three regression models (McCain 2007a): one linear (variables not transformed), another semilogarithmic (log‐transforming area), and another curvilinear (log‐transformed both area and species richness). We used the second‐order Akaike information criterion (AIC_c_) to select the best‐fitting model. We calculated area‐corrected diversity curves using a power function model (*S* = cA^*z*^) with a global taxon‐specific *z* value (slope of linear regressions) for those mountain ranges with significant species–area effects.

### Mid‐domain effect

We analyzed whether observed gamma‐diversity patterns fit those expected under mid‐domain hypothesis (Colwell and Hurtt 1994; Colwell et al. 2004) using the program Mid‐Domain Null (McCain 2004). This program uses a Monte Carlo procedure to simulate species richness curves based on range midpoints or empirical range sizes within the domain limits of the study. The empirical species richness curves were compared with the 95% predicted curves based on 50,000 simulations sampled without replacement from empirical species range sizes. The expected results were plotted against the empirical elevation richness to visually examine whether our observed results deviate from the null altitudinal range distribution. We tested the fit between the observed empirical values and the predicted number of species under the mid‐domain model (i.e., predicted richness and its 95% confidence interval) using both linear and quadratic regressions. We chose the model with the lowest AIC_c_ as the best‐fitting model. Sampling of simulations with replacement yielded similar results (not shown). The range of species known only from a single locality was increased ±5 m to provide a nonzero size range in our analysis, following Hutter et al. (2013). Because a SAR is expected to modify the predictions of mid‐domain model, we assessed whether the fit to this model improved when area effect was accounted (McCain 2007a).

### Spatial environmental heterogeneity effect

Some of the most used measures of SHE are topographic heterogeneity, diversity of land cover types, and plant species richness (Stein et al. 2014). Here, we use topographic heterogeneity to evaluate the interaction between SEH and species richness pattern of *Pristimantis* frogs. Topographic heterogeneity was calculated for each 500‐m‐wide altitudinal bands of Tropical Andes, each Andean domain, and each mountain range, using the topographic ruggedness index (TRI) developed by Riley et al. (1999). This index expresses the difference in elevation between neighborhood cells of a digital elevation grid. The TRI was calculated on the global digital elevation model (GTOPO30) using the function Ruggedness Index in the Terrain Analysis plugin under Quantum GIS software (QGIS Development Team, 2013). Topographic heterogeneity effect was evaluated using three linear regression models: linear (variables not transformed), semilogarithmic (log‐transformed TRI), and curvilinear (log‐transformed variables). As area is often related to SHE (Rosenzweig 1995), we repeated the topographic heterogeneity analysis accounting for area using as the dependent variable the TRI values divided by the squared root of area. We used AIC_c_ to select the best‐fitting model.

### Latitudinal effect

To estimate latitudinal trends, we calculated the mid latitudinal point and the average TRI for each mountain range from the global digital elevation model (GTOPO30) using Quantum GIS software (QGIS Development Team, 2013). Latitudinal effect was evaluated via linear regressions using data from species richness, mid‐elevational distributional point, mid latitudinal point, and average TRI for each main mountain range studied here. We evaluated four models, with species richness being explained by latitude (model 1), by latitude and altitude (model 2), by latitude and TRI (model 3), and an intercept‐only model (model 4). We estimated the parameters’ coefficient of these models using averaged modeling (Anderson 2008), implemented in the R package AICcmodavg (Mazerolle 2015). Unless otherwise indicated, all statistical analyses were performed using R (R Development Core Team 2013).

## Results

### Species richness pattern

Frogs of the genus *Pristimantis* were distributed over a large altitudinal range with the lowest altitudinal distribution in the lowest elevation limit (500 m) and the highest altitudinal distribution up to 4538 m. The highest diversity of species was concentrated in the North Andes (311 species) and drooped markedly in the Central Andes (100 species), with the lowest diversity in the Eastern Cordillera of Bolivian Andes (11 species). We found a hump‐shaped pattern in the tropical Andes and each of its domains and mountain ranges, except in the Bolivian Andes (Fig. 2, see Appendix S2, Fig. S1). The elevation of the richness peak varied among domains and mountain ranges. Richness peaked between 2000 and 3500 m in the Tropical and Northern Andes and between 1500 and 3000 m in the Central Andes. In the Eastern Cordillera of Bolivia, there was a low plateau pattern, with high species richness at lower elevations (500–2000 m.a.s.l.).

### Species–area relationship

Surface area did not always show a decreasing pattern with ascending elevations. The area of the Tropical and Central Andes (Fig. 2) domains decreased with increasing elevation up to 2500–3000 m, then increased to reach a peak at an elevation between 3500 m and then decreased at higher elevations (Fig. 2). In both cases, the peak in area above 3500 m of elevation coincided with the existence of high‐elevation plateau on the Peruvian and Bolivian Andes. In contrast, the area of the Northern Andes (Fig. 2) showed a decreasing pattern, where the area decreased monotonically with an increase of elevation. The area profiles on the main mountain ranges of the Northern Andes domain generally decreased with elevation, whereas the area in the mountain ranges of Central Andes showed a hump at high elevations (see Appendix S2, Fig. S1).

**Figure 3:**
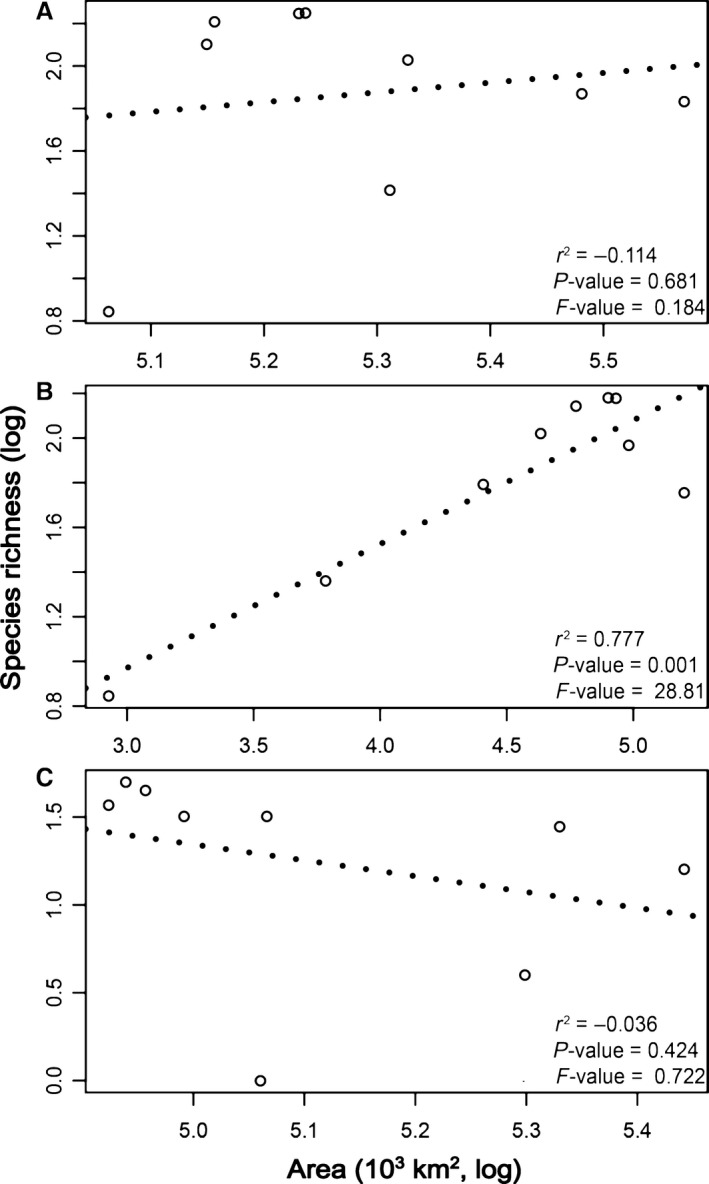
Species–area effects in elevational gradients of the Tropical (A), Northern (B), and Central Andes (C). Values inside each figure are results of simple linear regressions. All the *F*‐values used df = 1,7.

Surface area did not always show a positive correlation with species richness (Fig. 3). The curvilinear effect was the best‐fit model to SARs on the Tropical Andes and its domains (ΔAIC_c_ > 7). There was no relationship between area and species richness in the Tropical (*r* ^*2*^ = −0.114, *P*‐value = 0.681) and Central Andes (*r* ^*2*^ = −0.036, *P*‐value = 0.424). In contrast, a significant effect was found in the SAR along the altitudinal gradient in the Northern Andes (*r* ^*2*^ = 0.777, *P*‐value < 0.001). Similar results were recorded for all area–species relationships along the main Andean mountain ranges where *Pristimantis* frogs occur. In all cases, the curvilinear effect was always the best‐fit model of SARs. Significant curvilinear species–area effects were detected in five mountain ranges on the Northern Andes, with *r* ^*2*^ values ranging from 0.396 to 0.740. Nonsignificant relationship between diversity and area (*P*‐value > 0.05) was detected along each main mountain range of the Central Andes (see Appendix S1, Table S2).

**Figure 4:**
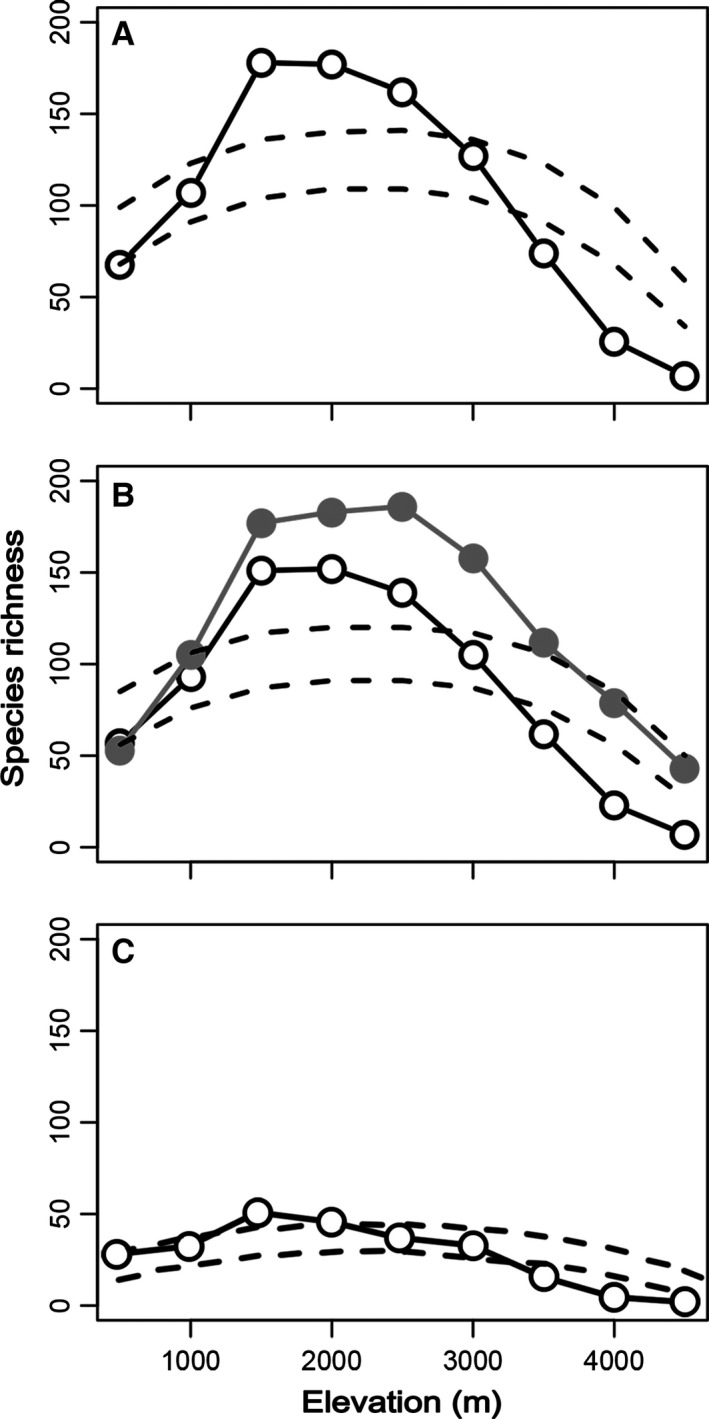
Hump‐shaped patterns in species richness for Pristimantis frogs along elevational gradients of the Tropical (A), Northern (B), and Central Andes (C). The 95% confidence intervals generated from the mid‐domain null model plotted for comparison (dashed lines). Gray line in B indicates the curvilinear area‐corrected richness pattern.

Curvilinear regressions to calculate global taxon‐specific z values for correcting area effects give a global *z* value of 0.36 with 95% confidence limits of 0.18–0.54 for *Pristimantis* frogs. Correcting for curvilinear area effects had little impact in the shape of the empirical pattern of biodiversity curves. The shape of the corrected pattern of species richness along the altitudinal gradient in the Northern Andes was very similar to the empirical pattern with the diversity peak located at high elevations (Fig. 4B). Similarly, in each main mountain range where significant curvilinear species–area effects were detected, the diversity patterns showed no change in the location of the diversity peak (see Appendix S2, Fig. S2).

### Mid‐domain effect

The MDE analysis produced similar gradients in species richness relative to the empirical gradients, but the fit of the model varied among regions (Fig. 4). We identified MDE as a good predictor of species richness in all the Tropical Andes and in the Northern Andes domain, whereas the explanatory power of the model was moderate in the Central Andes domain. The good fit to the null model predictions in the Tropical and Northern Andes was demonstrated by the high *r* ^*2*^ values (0.86 and 0.87, respectively, *P*‐value < 0.001). In contrast, moderate *r* ^*2*^ value was observed in the Central Andes (0.65, *P*‐value 0.005). Quadratic and linear models gave similar results based on *r* ^*2*^ and AIC_c_ values (Table 1). Deviations from the null model occurred at mid‐elevations and highest elevations for the Tropical Andes and the two domains. The fit to spatial constraints was highly variable in each mountain range studied here. The quadratic regression was the best model (ΔAIC_c_ = 5.5) for area‐corrected diversity curves with MDE predictions for the Northern Andes. This model improved the resulting fit of the model with an *r* ^*2*^ value of 0.9 (*P*‐value < 0.001). However, spatial constrain fitting was variable in each mountain range studied here, even when we accounted for species–area effects (see Appendix S1, Table S3).

**Table 1:** Explanatory power of spatial constraint effects (MDE) using linear and quadratic regressions statistics

Geographic region	Model	*F*_(1,7)_	*P*‐value	*r* ^2^	AIC_c_
Tropical Andes	Linear	31.42	0.0008	0.79	73.9
	Quadratic	27.51	0.0001	0.86	70.4
Northern Andes	Linear	55.16	0.0001	0.87	84.1
	Quadratic	24.42	0.0013	0.85	85.4
Central Andes	Linear	15.85	0.0053	0.65	56.6
	Quadratic	8.17	0.0194	0.64	57.5

### Spatial environmental heterogeneity effect

The relationships between topographic heterogeneity and species richness on the Tropical Andes and its domains were best fit by curvilinear models (Appendix S1, Table S4). There was no relationship between TRI and species richness in the Tropical (*r* ^*2*^ = −0.1349, *P*‐value = 0.831) and Central Andes (*r* ^*2*^ = 0.018, *P*‐value = 0.320), but a significant effect was found in the Northern Andes (*r* ^*2*^ = 0.366, *P*‐value = 0.049). Similarly, a curvilinear effect was the best‐fit model in the main Andean mountain ranges studied here. Significant effects were detected in three mountain ranges on the Northern Andes, with *r* ^*2*^ values ranging from 0.767 to 0.847. In contrast, in the Central Andes, only the Eastern Cordillera of Peru showed a significant effect (*r* = 0.445; *P*‐value = 0.030).

### Latitudinal effects

We found a negative relationship between species richness and latitude in the three models (models 1, 2, and 3). The models with the best fit were model 1 (species richness explained by latitude) and model 3 (species richness explained by latitude plus spatial topographic heterogeneity), which differ by a ΔAIC_c_ of 0.94. Based on average modeling values of the three proposal models, latitude was the most important parameter explaining species richness of *Pristimantis* frogs (model‐averaged estimate = −4.55) in comparison with topography heterogeneity (model‐averaged estimate = −0.07) and elevation (model‐averaged estimate = 0.01).

## Discussion

The Tropical Andes harbor an extraordinary number of species, but a detailed picture of the spatial distribution of this biodiversity along altitudinal and latitudinal gradients is still incipient (Mutke et al. 2014). Many studies have documented that species richness along Andean elevational gradients generally follows a hump‐shaped pattern with the highest richness at some mid‐elevational point. Recent evidence suggests that historical and ecological processes are the major drivers of this pattern in the Andes (Hutter et al. 2013; Castroviejo‐Fisher et al. 2014). However, the effect of spatial factors (SAR and MDE) on such diversity pattern has rarely been considered. Here, we found that in *Pristimantis* frogs, the hump‐shaped richness pattern is consistent across multiple mountain ranges, even when accounting for area. We also found that in some Andean elevational gradients, MDE seem to be a good predictor of species richness patterns, but the fit to the model varied among mountain ranges. Our findings suggest that spatial factors are partly linked to biodiversity patterns, but are not the only driving mechanism. Other possible drivers for this species richness pattern are discussed below.

### Species richness pattern

The richness of several Tropical Andes clades reaches its diversity peak at intermediate elevations (e.g., land birds: Rahbek 1997; Kattan and Franco 2004; McCain 2009; mammals: McCain 2007a, 2007b; glassfrogs: Hutter et al. 2013; ferns: Karger et al. 2011; Salazar et al. 2015). This spatial pattern has also been observed in several clades from many other mountain regions around the world (e.g., treefrogs of Middle America: Smith et al. 2007; salamanders of Middle America: Wiens et al. 2007; fishes of Tibetan Plateau: Li et al. 2009; salamanders of North America: Kozak and Wiens 2010; birds of Himalaya: Acharya et al. 2011; among others). *Pristimantis* frogs generally showed a hump‐shaped pattern with the highest richness at mid‐elevations in the Tropical Andes. This pattern was consistent along each Andean mountain range studied here except for the Bolivian Andes, where we observed a low plateau pattern, with the high species richness at lower elevations.

The only other elevation gradient of diversity for the Bolivian Andes, regarding dung beetles (Herzog et al. 2013), shows a distribution pattern with a peak of highest richness between 250 and 499 m.a.s.l., similar to what we observed in *Pristimantis*. The low plateau pattern in Bolivia could be associated with contemporary climatic factors (e.g., temperature, productivity, and water availability), which have been proposed to influence elevational biodiversity patterns (see below). Alternatively, due the arid and semiarid climatic conditions in most highlands of the Bolivian Andes (Garreaud et al. 2003), we hypothesize a higher rate of extinction and lower rate of speciation on this area relative to humid Andean lowlands adjacent to the Amazonia. Furthermore, the retention of ancestral climatic tolerances (niche conservatism hypothesis) could have constrained the current geographic distribution of most lowland species, as suggested by Herzog et al. (2013). Additional analyses of elevational diversity in the Bolivian Andes will help to the understanding of the mechanism driving this pattern of biodiversity.

### Species–area relationship

Area is an important factor to explain species richness patterns along elevational gradients because different altitudinal bands have different areas (Körner 2000; Sanders 2002; McCain and Grytnes 2010). On mountains, area usually declines with increasing elevation and, as a result, gamma‐diversity tends to follow the same pattern (Rosenzweig 1995; Rahbek 1997; Lomolino 2001; McCain 2007a). However, in large and complex mountain systems, such as the Tropical Andes, relief variation influences elevational belt areas, resulting in area profiles that do not follow a uniform pattern.

Our results show that area influences richness patterns of *Pristimantis* frogs in the Tropical Andes. In 45% of the mountain ranges studied here, area was related to the elevational pattern in species richness (see Appendix S1, Table S2). Interestingly, the area effect was more pronounced in the Northern Andes, where 71% of the elevational gradients of species richness showed strong responses to area. This effect could be associated with the fact that in the Northern Andes, area generally decreases with elevation, which leads to strong SARs (see McCain 2007a). In contrast, in the Central Andes, area showed a peak at high elevations coinciding with the extensive areas of altiplano in highlands, resulting in negative or nonsignificant relationships between diversity and area.

The high variability in the response of elevational diversity to area indicates that it influences species richness patterns of *Pristimantis* frogs, but it is not the main driver of the observed curves of diversity. Similar responses have also been reported in previous analyses of several mountain systems (McCain 2007a; Karger et al. 2011). Such results suggest that area could represent a source of error if is not properly accounted for in the analyses, but it is not the sole explanatory mechanism of the observed curves of biodiversity (McCain 2007a).

### Mid‐domain effect

Despite the shape of the empirical biodiversity curves deviating from the MDE prediction, regressions analysis (linear and quadratic) showed that this model explains an important proportion of the altitudinal patterns of *Pristimantis* diversity in the Tropical and Northern Andes. Spatial constraints around main mountain ranges studied here were also highly variable (see Appendix S1, Table S3). In fact, only 45% of the elevational gradients were consistent with MDE predictions. Previous analyses suggested that the SAR influencing the MDE fit in several degrees (McCain 2005, 2007a). Some studies have found significant increases in MDE fit (Sanders 2002; Bachman et al. 2004) when area effect was accounted for, whereas others found no large improvements or even decreases (McCain 2005, 2007a, 2009). We found that the fits to MDE vary when area effect was included in the model, being improved in some cases, but worsened in others. After the area effect in the model was included, only one gradient fits with MDE (see Appendix S1, Table S3), supporting the idea that area is an important factor that should be taken into consideration in the spatial analysis of diversity (see McCain 2007a).

### Spatial environmental heterogeneity effect

Our analysis indicates that topographic heterogeneity effects on species richness of *Pristimantis* frogs differ spatially. We did not find any significant relationship between topographic heterogeneity and species richness in Tropical Andes (as a whole) or in the Central Andes domain. Remarkably, topographic effects were more pronounced in the Northern Andes domain, where topographic heterogeneity explains partially the observed pattern of species richness in this domain and three of its mountain ranges (Appendix S1, Table S4). This positive relationship has been related to the fact that highly heterogeneous regions provide more long‐term stable niches to support more species than regions of lower heterogeneity (Rosenzweig 1995; Thuiller et al. 2006; Allouche et al. 2012; Stein et al. 2014). The absence of topographic heterogeneity effects in southern latitudes (i.e., Central Andes domain and most of its mountain ranges) may be due to the strong influence of climatic seasonality of the Andes south of the Equator, a recognized factor limiting the occurrence of tropical species. Although our results shown that topographic heterogeneity is in some cases a good predictor of species richness patterns of *Pristimantis* frogs, the high level of variation found in our analysis suggests that other factors are also important driving for species diversity. Further studies may help to understand whether other components of SEH (e.g., land cover types, vegetation diversity, and soil type, among others) also explain the species richness patterns in montane anurans.

### Latitudinal effect

The highest diversity of the genus *Pristimantis* was found in latitudes slightly north of the equatorial line and decreased in northern (Sierra Nevada de Santa Marta in Colombia and Meridian Andes in Venezuela) and southern (Bolivian Andes) latitudes. In one of the few studies on the latitudinal gradient of biodiversity in the Tropical Andes, a similar latitudinal pattern was found in eastern Andean Lepidoptera species. However, in these butterflies and moths, the peak of highest richness is reached at southern latitudes between the Huancabamba depression and central Peru (Pyrcz et al. 2013). In Lepidoptera species, the latitudinal gradient has been explained as a result of greater area, age of the southern tropical Andes, and seasonal temperatures of the Andes south of the Equator (Pyrcz et al. 2013). However, as the highest richness of *Pristimantis* frogs was found in the northern tropical Andes, we consider that area and geological age may not represent the main factors shaping the latitudinal diversity of the genus.

The dramatic decrease in species richness in the Bolivian Andes has been observed in other taxa (birds: Rahbek and Graves 2001; insects: Pyrcz et al. 2013). This phenomenon has been related to increased seasonality in southern Bolivia, which has been recognized as a crucial limiting factor for tropical species (Pyrcz and Gareca 2009; Pyrcz et al. 2013). Our data also suggest that in the western Andes, there is higher species richness in northern rather than in southern latitudes among *Pristimantis* frogs; further analyses on other taxa may reveal whether this is a common pattern and which mechanisms are shaping latitudinal patterns of species richness in Andean organisms.

### Climatic drivers

Several ongoing climatic factors (such as temperature, productivity, and precipitation) have been proposed to influence elevational biodiversity patterns in a wide range of organisms along Andean elevational gradients (birds: Terborgh 1977; McCain 2009; bats: McCain 2007b; epiphytes: Krömer et al. 2005). However, few studies have investigated the role of these variables explaining elevational patterns of species richness among Andean amphibians. Recent evidence from the Antioquia department in the Central Cordillera of Colombia shows a high correlation between amphibian species richness and temperature and precipitation (Ortiz‐Yusty et al. 2013). An analysis of this kind, extended to the Andes Mountains, might indicate whether climatic factors are also critical to explaining the diversity of *Pristimantis*. The fact that *Pristimantis* frogs are restricted principally to moist forest habitats (Lynch and Duellman 1997; Pinto‐Sánchez et al. 2012) suggests that a combination of climatic optimal conditions and local environmental features play an important role in shaping the species richness patterns. Further studies to examine the relationship between species richness and climatic variables should compile climatic data estimates per altitudinal band in Tropical Andes, a piece of information currently unavailable. Such data could be analyzed using regression analysis models (e.g., ordinary least squares, generalized least squares, among others) and have the potential to shed more light on how climate variables are important in shaping diversity curves in rain frogs and other taxa.

### Evolutionary history

Recent evidence from Andean glassfrogs suggests that evolutionary processes, in particular greater time for speciation at mid‐elevations (the “montane museum hypothesis”), have considerably shaped their current diversity patterns (Hutter et al. 2013; Castroviejo‐Fisher et al. 2014). Evidence from other anuran Andean clades (poison‐dart frogs) supports the old origin of the group at mid‐Andean elevations and subsequent long‐term diversification (Santos et al. 2009). Additional support for this hypothesis from other montane regions around the world includes Middle American treefrogs (Smith et al. 2007), plethodontid salamanders (Wiens et al. 2007), Tibetan fishes (Li et al. 2009), and Appalachian plethodontid salamanders (Kozak and Wiens 2010). In addition, climatic‐niche conservatism underlies the montane museum hypothesis and explains the hump‐shaped pattern of species richness in glassfrogs (Hutter et al. 2013). On the contrary, evidence from the anuran clade Terrarana, which includes the *Pristimantis* genus, contradicts the montane museum hypothesis based on the fact that older clades have less species than recent ones (Gonzalez‐Voyer et al. 2011). To test this interesting and promising hypothesis in *Pristimantis,* we need a more densely sampled (including at least 30% of species) phylogeny than those currently available.

## Conflict of Interest

None declared.

## Supporting information


**Appendix S1**. Supporting tables.
**Table S1.** For each species, we provide the minimum, maximum, midpoint, and range size of elevation (meters above sea level).
**Table S2.** Species‐area effect using non‐transformed variables (linear effect), log‐transformed variables (curvilinear effect), and log‐transformed area (semi‐log effect).
**Table S3.** Fitting of the spatial constraint effects model (MDE) for empirical and corrected biodiversity curves using linear and quadratic regressions statistics for each main mountain ranges studied here.
**Table S4.** Effect of spatial topographic heterogeneity effect on species richness using non‐transformed variables (linear effect), log‐transformed variables (curvilinear effect), and log‐transformed area (semi‐log effect).
**Appendix S2**. Supporting figures.
**Figure S1.** Area profiles (open squares and dotted lines) and diversity pattern (solid circles and solid lines) along elevational gradients on main mountain ranges of Tropical Andes.
**Figure S2.** Comparisons among curvilinear area correction method (solid circles and solid lines) and empirical diversity patterns (open squares and dotted lines) for each main mountain ranges where significant curvilinear species‐area effects were detected.
