# Supplementary material for: Spatial diversity patterns of Pristimantis frogs in the Tropical Andes
Source: Ecol Evol. 2016 Feb 19;6(7):1901–13. doi: 10.1002/ece3.1968 (PMC4759521; doi:10.1002/ece3.1968)
Supplement: Supplementary file 1 — Appendix S1. Supporting tables. Table S1. For each species, we provide the minimum, maximum, midpoint, and range size of elevation (meters above sea level). Table S2. Species‐area effect using non‐transformed variables (linear effect), log‐transformed variables (curvilinear effect), and log‐transformed area (semi‐log effect). Table S3. Fitting of the spatial constraint effects model (MDE) for empirical and corrected biodiversity curves using linear and quadratic regressions statistics for each main mountain ranges studied here. Table S4. Effect of spatial topographic heterogeneity effect on species richness using non‐transformed variables (linear effect), log‐transformed variables (curvilinear effect), and log‐transformed area (semi‐log effect). Appendix S2. Supporting figures. Figure S1. Area profiles (open squares and dotted lines) and diversity pattern (solid circles and solid lines) along elevational gradients on main mountain ranges of Tropical Andes. Figure S2. Comparisons among curvilinear area correction method (solid circles and solid lines) and empirical diversity patterns (open squares and dotted lines) for each main mountain ranges where significant curvilinear species‐area effects were detected. [file ECE3-6-1901-s001.docx]

**Supporting Information**

**Spatial diversity patterns of Pristimantis frogs in the Tropical Andes**

Fabio Leonardo Meza-Joya and Mauricio Torres

**Appendix S1. Supporting tables**

**Table S1.** For each species, we provide the minimum, maximum, midpoint, and range size of elevation (meters above sea level). If a species occurs in the Andes and adjacent lowlands we assumed its potential elevational range from the minimum non-Andean elevation to the maximum Andean elevation. Species with distributional range out of Andes were excluded from our analyses.

| **Species** | **Minimum** | **Maximum** | **Midpoint** | **Range Size** | **Reference** |
| --- | --- | --- | --- | --- | --- |
| *P. acatallelus* | 1410 | 2600 | 2005 | 1190 | Ruiz-Carranza *et al*. (1997); Frost (2013); IUCN (2013) |
| *P. acerus* | 2660 | 2750 | 2705 | 90 | Lynch & Duellman (1980); Frost (2013); IUCN (2013) |
| *P. achatinus* | 0 | 2600 | 1300 | 2600 | Frost (2013); IUCN (2013); Rojas *et al*. (2013) |
| *P. actinolaimus* | 1800 | 2000 | 1900 | 200 | Galvis-Peñuela & Rueda-Almonacid (2004) |
| *P. actites* | 760 | 2486 | 1623 | 1726 | Frost (2013); IUCN (2013) |
| *P. acuminatus* | 100 | 900 | 500 | 800 | Frost (2013); IUCN (2013) |
| *P. acutirostris* | 1740 | 2400 | 2070 | 660 | Frost (2013); IUCN (2013) |
| *P. adiastolus* | 1200 | 1200 | 1200 | 0 | Duellman & Hedges (2007); IUCN (2013) |
| *P. aemulatus* | 1410 | 1430 | 1420 | 20 | Ruiz-Carranza *et al*. (1997); Frost (2013) |
| *P. affinis* | 2600 | 3300 | 2950 | 700 | Frost (2013); IUCN (2013) |
| *P. alalocophus* | 2650 | 3100 | 2875 | 450 | Frost (2013); IUCN (2013) |
| *P. albericoi* | 950 | 950 | 950 | 0 | Ruiz-Carranza *et al*. (1997); Frost (2013); IUCN (2013) |
| *P. albertus* | 1970 | 1970 | 1970 | 0 | Duellman & Lehr (2009); Frost (2013); IUCN (2013) |
| *P. altamnis* | 400 | 1000 | 700 | 600 | Elmer & Cannatella (2008); Frost (2013); IUCN (2013) |
| *P. ameliae* | 2500 | 2500 | 2500 | 0 | Barrio-Amorós (2011); Frost (2013) |
| *P. amydrotus* | 1500 | 1500 | 1500 | 0 | Duellman & Lehr (2007, 2009); Frost (2013); IUCN (2013) |
| *P. andinognomus* | 2400 | 2800 | 2600 | 400 | Lehr & Coloma (2008) |
| *P. anemerus* | 2770 | 2770 | 2770 | 0 | Duellman & Pramuk (1999); Frost (2013) |
| *P. angustilineatus* | 1880 | 2500 | 2190 | 620 | Frost (2013); IUCN (2013) |
| *P. aniptopalmatus* | 2300 | 2600 | 2450 | 300 | Duellman & Lehr (2009); Frost (2013); IUCN (2013) |
| *P. anolirex* | 1900 | 3550 | 2725 | 1650 | Stuart *et al*. (2008); Frost (2013); IUCN (2013) |
| *P. apiculatus* | 1750 | 2120 | 1935 | 370 | Frost (2013); IUCN (2013) |
| *P. appendiculatus* | 1460 | 2800 | 2130 | 1340 | Frost (2013); IUCN (2013) |
| *P. aquilonaris* | 2000 | 2500 | 2250 | 500 | Lehr *et al*. (2007); Duellman & Lehr (2009); IUCN (2013) |
| *P. ardalonychus* | 680 | 1200 | 940 | 520 | Lehr *et al*. (2007); Duellman & Lehr (2009); IUCN (2013) |
| *P. atrabracus* | 2963 | 3330 | 3147 | 367 | Duellman & Pramuk (1999); Lehr *et al*. (2007); Frost (2013) |
| *P. atratus* | 2195 | 2850 | 2523 | 655 | Lynch (1979); Frost (2013); IUCN (2013) |
| *P. aurantiguttatus* | 1000 | 1900 | 1450 | 900 | Frost (2013); IUCN (2013) |
| *P. avicuporum* | 1700 | 2030 | 1865 | 330 | Frost (2013); IUCN (2013) |
| *P. bacchus* | 1314 | 2300 | 1807 | 986 | Frost (2013); IUCN (2013); MHUA (2013) |
| *P. baiotis* | 1780 | 2000 | 1890 | 220 | Lynch (1998); Frost (2013); IUCN (2013) |
| *P. balionotus* | 2800 | 2800 | 2800 | 0 | Lynch (1979); Frost (2013); IUCN (2013) |
| *P. bambu* | 2876 | 2989 | 2933 | 113 | Arteaga-Navarro & Guayasamin (2011); Frost (2013) |
| *P. baryecuus* | 2195 | 2988 | 2592 | 793 | Lynch (1979); Frost (2013); IUCN (2013) |
| *P. batrachites* | 2180 | 2250 | 2215 | 70 | Lynch (2003a); Frost (2013); IUCN (2013) |
| *P. bearsei* | 500 | 730 | 615 | 230 | Dulleman (1992); Duellman & Lehr (2009); Frost (2013) |
| *P. bellae* | 1800 | 2300 | 2050 | 500 | Reyes-Puig & Yánez-Muñoz (2012) |
| *P. bellator* | 1900 | 3100 | 2500 | 1200 | Lehr *et al*. (2007); Duellman & Lehr (2009); Frost (2013) |
| *P. bellona* | 1100 | 2000 | 1550 | 900 | Frost (2013); IUCN (2013) |
| *P. bernali* | 2350 | 2350 | 2350 | 0 | Lynch (1986); Frost (2013); IUCN (2013) |
| *P. bicantus* | 2100 | 2300 | 2200 | 200 | Guayasamin & Funk (2009) |
| *P. bicolor* | 1750 | 2400 | 2075 | 650 | Rueda-Almonacid & Lynch (1983); Frost (2013); IUCN (2013) |
| *P. bipunctatus* | 230 | 2320 | 1275 | 2090 | Lehr *et al*. (2006); Duellman & Lehr (2009); Frost (2013) |
| *P. boconoensis* | 2700 | 3150 | 2925 | 450 | Stuart *et al*. (2008); Frost (2013) |
| *P. bogotensis* | 2600 | 3400 | 3000 | 800 | Frost (2013) |
| *P. boulengeri* | 2520 | 2920 | 2720 | 400 | Lynch (1981); Frost (2013) |
| *P. brevifrons* | 1140 | 2610 | 1875 | 1470 | Lynch (1981, 1998); IUCN (2013) |
| *P. briceni* | 1600 | 3300 | 2450 | 1700 | Stuart *et al*. (2008); Frost (2013) |
| *P. bromeliaceus* | 1500 | 2622 | 2061 | 1122 | Duellman & Lehr (2009); Frost (2013); IUCN (2013) |
| *P. buckleyi* | 2400 | 3700 | 3050 | 1300 | Mueses-Cisneros (2005); Frost (2013); IUCN (2013) |
| *P. bustamante* | 2745 | 3016 | 2881 | 271 | Chaparro *et al*. (2012); Frost (2013) |
| *P. cabrerai* | 1140 | 1940 | 1540 | 800 | Stuart *et al*. (2008); Frost (2013); IUCN (2013) |
| *P. cacao* | 2190 | 2600 | 2395 | 410 | Lynch (1998); Frost (2013); IUCN (2013) |
| *P. caeruleonotus* | 2500 | 2900 | 2700 | 400 | Lehr *et al*. (2007); Duellman & Lehr (2009); Frost (2013) |
| *P. cajamarcensis* | 1800 | 3100 | 2450 | 1300 | Duellman & Lehr (2009); IUCN (2013) |
| *P. calcaratus* | 1400 | 2700 | 2050 | 1300 | Frost (2013); IUCN (2013) |
| *P. calcarulatus* | 1140 | 2700 | 1920 | 1560 | Stuart *et al*. (2008); Frost (2013); IUCN (2013) |
| *P. capitonis* | 2440 | 2800 | 2620 | 360 | Lynch (1998); Frost (2013); IUCN (2013) |
| *P. caprifer* | 50 | 950 | 500 | 900 | Frost (2013); IUCN (2013) |
| *P. carlossanchezi* | 2400 | 2550 | 2475 | 150 | Arroyo (2007); Frost (2013); IUCN (2013) |
| *P. carmelitae* | 1520 | 2200 | 1860 | 680 | Frost (2013); IUCN (2013) |
| *P. carranguerorum* | 1350 | 2060 | 1705 | 710 | Lynch (1994a); Frost (2013); IUCN (2013) |
| *P. caryophyllaceus* | 0 | 1500 | 750 | 1500 | IUCN (2013) |
| *P. celator* | 1780 | 2600 | 2190 | 820 | Lynch (1998); Frost (2013); IUCN (2013) |
| *P. ceuthospilus* | 1500 | 1840 | 1670 | 340 | Duellman & Lehr (2009); Frost (2013); IUCN (2013) |
| *P. chalceus* | 50 | 1970 | 1010 | 1920 | Lynch & Duellman (1997); Frost (2013); IUCN (2013) |
| *P. chimu* | 3000 | 3100 | 3050 | 100 | Lehr (2007), Duellman & Lehr (2009); Frost (2013); IUCN (2013) |
| *P. chloronotus* | 2285 | 3350 | 2818 | 1065 | Lynch & Duellman (1980); Frost (2013); IUCN (2013) |
| *P. chrysops* | 900 | 2200 | 1550 | 1300 | Frost (2013); IUCN (2013) |
| *P. citriogaster* | 600 | 1094 | 847 | 494 | Camacho-Badani *et al*. (2012); Frost (2013) |
| *P. colodactylus* | 2195 | 3140 | 2668 | 945 | Duellman & Lehr (2009); Frost (2013); IUCN (2013) |
| *P. colomai* | 830 | 1200 | 1015 | 370 | Lynch & Duellman (1997); Stuart *et al*. (2008); IUCN (2013) |
| *P. colonensis* | 2200 | 2750 | 2475 | 550 | Mueses-Cisneros (2007); Yánez-Muñoz *et al*. (2012) |
| *P. colostichos* | 3000 | 3600 | 3300 | 600 | La Marca & Smith (1982); Stuart *et al*. (2008); IUCN (2013) |
| *P. condor* | 1500 | 1975 | 1738 | 475 | Duellman & Lehr (2009); Frost (2013) |
| *P. conservatio* | 1640 | 1640 | 1640 | 0 | Barrio-Amorós *et al*. (2013) |
| *P. conspicillatus* | 0 | 600 | 300 | 600 | Duellman & Lehr (2009); Frost (2013); IUCN (2013) |
| *P. cordovae* | 3400 | 3642 | 3521 | 242 | Duellman & Lehr (2007, 2009); Frost (2013); IUCN (2013) |
| *P. corniger* | 1500 | 2600 | 2050 | 1100 | Lynch & Suárez-Mayorga (2003); IUCN (2013) |
| *P. coronatus* | 2850 | 2850 | 2850 | 0 | Duellman & Lehr (2009); Frost (2013); IUCN (2013) |
| *P. corrugatus* | 3000 | 3300 | 3150 | 300 | Duellman & Lehr (2009); Frost (2013); IUCN (2013) |
| *P. cosnipatae* | 1570 | 1800 | 1685 | 230 | Duellman & Lehr (2009); Frost (2013); IUCN (2013) |
| *P. cremnobates* | 1410 | 1700 | 1555 | 290 | Lynch & Duellman (1980); Frost (2013); IUCN (2013) |
| *P. crenunguis* | 760 | 2486 | 1623 | 1726 | Stuart *et al*. (2008); Frost (2013); IUCN (2013) |
| *P. cristinae* | 1530 | 3500 | 2515 | 1970 | Rueda-Solano & Vargas-Salinas (2010); Frost (2013) |
| *P. crucifer* | 1200 | 1800 | 1500 | 600 | Stuart *et al*. (2008); Frost (2013); IUCN (2013) |
| *P. cruciocularis* | 1330 | 1850 | 1590 | 520 | Lehr *et al*. (2006); Duellman & Lehr (2009); IUCN (2013) |
| *P. cruentus* | 200 | 805 | 503 | 605 | Frost (2013) |
| *P. cryophilius* | 2835 | 3384 | 3110 | 549 | Stuart *et al*. (2008); Frost (2013); IUCN (2013) |
| *P. cryptomelas* | 2470 | 3100 | 2785 | 630 | Duellman & Lehr (2009); Frost (2013); IUCN (2013) |
| *P. cuentasi* | 2800 | 2800 | 2800 | 0 | Lynch (2003b); Frost (2013); IUCN (2013) |
| *P. culatensis* | 2870 | 2900 | 2885 | 30 | La Marca (2007); IUCN (2013) |
| *P. cuneirostris* | 1700 | 1700 | 1700 | 0 | Duellman & Lehr (2009); Frost (2013); IUCN (2013) |
| *P. curtipes* | 2750 | 4400 | 3575 | 1650 | Frost (2013); IUCN (2013) |
| *P. danae* | 500 | 1850 | 1750 | 1350 | Duellman & Lehr (2009); Frost (2013); Chaparro, J.C. (pers. comm.) |
| *P. degener* | 830 | 1200 | 1015 | 370 | Lynch & Duellman (1997); Frost (2013); IUCN (2013) |
| *P. deinops* | 1750 | 2600 | 2175 | 850 | Lynch (1998); Frost (2013); IUCN (2013) |
| *P. devillei* | 2350 | 3150 | 2750 | 800 | Frost (2013); IUCN (2013) |
| *P. diadematus* | 0 | 1150 | 575 | 1150 | Duellman & Lehr (2009); IUCN (2013) |
| *P. diaphonus* | 1180 | 1250 | 1215 | 70 | Lynch (1998); Frost (2013); IUCN (2013) |
| *P. diogenes* | 1470 | 1600 | 1535 | 130 | Lynch (1998); Frost (2013); IUCN (2013) |
| *P. dissimulatus* | 1920 | 2020 | 1970 | 100 | Lynch & Duellman (1997); Stuart *et al*. (2008); IUCN (2013) |
| *P. dorsopictus* | 2400 | 3000 | 2700 | 600 | Stuart *et al*. (2008); Frost (2013); IUCN (2013) |
| *P. douglasi* | 1800 | 2550 | 2175 | 750 | Stuart *et al*. (2008); Frost (2013) |
| *P. duellmani* | 1780 | 2700 | 2240 | 920 | Stuart *et al*. (2008); Frost (2013); IUCN (2013) |
| *P. duende* | 3450 | 3450 | 3450 | 0 | Lynch (2001); Frost (2013); IUCN (2013) |
| *P. elegans* | 2600 | 3300 | 2950 | 700 | Stuart *et al*. (2008); Frost (2013); IUCN (2013) |
| *P. epacrus* | 740 | 1660 | 1200 | 920 | Lynch & Suárez-Mayorga (2000); IUCN (2013) |
| *P. eremitus* | 1540 | 2100 | 1820 | 560 | Stuart *et al*. (2008); Frost (2013); IUCN (2013) |
| *P. eriphus* | 2160 | 2750 | 2455 | 590 | Guayasamin & Funk (2009); Frost (2013); IUCN (2013) |
| *P. ernesti* | 3900 | 3900 | 3900 | 0 | Stuart *et al*. (2008); Frost (2013); IUCN (2013) |
| *P. erythropleura* | 980 | 2600 | 1790 | 1620 | Ruiz-Carranza *et al*. (1997); Frost (2013); IUCN (2013) |
| *P. eugeniae* | 1700 | 2010 | 1855 | 310 | Lynch & Duellman (1997); Frost (2013); IUCN (2013) |
| *P. exoristus* | 665 | 1830 | 1248 | 1165 | Duellman & Lehr (2009); Frost (2013); IUCN (2013) |
| *P. factiosus* | 1800 | 2200 | 2000 | 400 | Lynch & Rueda-Almonacid (1998a); IUCN (2013) |
| *P. fallax* | 1100 | 1850 | 1475 | 750 | Lynch & Rueda-Almonacid (1999); IUCN (2013) |
| *P. fasciatus* | 800 | 1200 | 1000 | 400 | Barrio-Amorós *et al*. (2007); IUCN (2013) |
| *P. fenestratus* | 100 | 1800 | 950 | 1700 | Duellman & Lehr (2009); Frost (2013) |
| *P. festae* | 2360 | 4400 | 3380 | 2040 | Frost (2013); IUCN (2013) |
| *P. fetosus* | 1800 | 2650 | 2225 | 850 | Lynch & Rueda-Almonacid (1998a); Frost (2013) |
| *P. flabellidiscus* | 2860 | 2920 | 2890 | 60 | La Marca (2007) |
| *P. flavobracatus* | 1770 | 1770 | 1770 | 0 | Lehr *et al*. (2006); Duellman & Lehr (2009); IUCN (2013) |
| *P. floridus* | 700 | 2000 | 1350 | 1300 | Lynch & Duellman (1997); Duellman & Lehr (2009) |
| *P. frater* | 1000 | 1600 | 1300 | 600 | Frost (2013); IUCN (2013) |
| *P. gagliardoi* | 2876 | 2989 | 2933 | 113 | Bustamante & Mendelson (2008); Frost (2013) |
| *P. gaigeae* | 150 | 1200 | 675 | 1050 | Ruiz-Carranza *et al*. (1996); IUCN (2013) |
| *P. galdi* | 1000 | 2250 | 1625 | 1250 | Duellman & Lehr (2009); IUCN (2013) |
| *P. ganonotus* | 1700 | 2000 | 1850 | 300 | Duellman & Lynch (1988); Frenkel *et al*. (2011) |
| *P. ginesi* | 2900 | 4000 | 3450 | 1100 | IUCN (2013); La Marca (2007) |
| *P. gentryi* | 2850 | 3380 | 3115 | 530 | Lynch & Duellman (1997); Frost (2013); IUCN (2013) |
| *P. gladiator* | 2270 | 2910 | 2590 | 640 | Mueses-Cisneros (2005); Guayasamin & Funk (2009) |
| *P. glandulosus* | 2105 | 2980 | 2543 | 875 | Stuart *et al*. (2008); Frost (2013); IUCN (2013) |
| *P. gracilis* | 1430 | 2740 | 2085 | 1310 | Lynch (1986); Lynch (1998) |
| *P. grandiceps* | 2200 | 2400 | 2300 | 200 | Ruiz-Carranza *et al*. (1996); Frost (2013); IUCN (2013) |
| *P. gryllus* | 900 | 2020 | 1460 | 1120 | Barrio-Amorós *et al*. (2012) |
| *P. hamiotae* | 2140 | 2140 | 2140 | 0 | Lynch &Duellman (1997); Frost (2013); IUCN (2013) |
| *P. hectus* | 1200 | 2020 | 1610 | 820 | Lynch & Duellman (1997); Frost (2013); IUCN (2013) |
| *P. helvolus* | 1800 | 2000 | 1900 | 200 | Stuart *et al*. (2008); Frost (2013); IUCN (2013) |
| *P. hernandezi* | 2300 | 2600 | 2450 | 300 | Frost (2013) |
| *P. huicundo* | 3229 | 3700 | 3465 | 471 | Guayasamin *et al*. (2004); Frost (2013); IUCN (2013) |
| *P. hybotragus* | 300 | 920 | 610 | 620 | Lynch (1998); IUCN (2013) |
| *P. ignicolor* | 2160 | 2750 | 2455 | 590 | Lynch & Duellman (1980); Frost (2013); IUCN (2013) |
| *P. illotus* | 1380 | 2560 | 1970 | 1180 | Stuart *et al*. (2008); Frost (2013); IUCN (2013) |
| *P. incanus* | 1700 | 2200 | 1950 | 500 | Stuart *et al*. (2008); IUCN (2013) |
| *P. incomptus* | 1370 | 1910 | 1640 | 540 | Stuart *et al*. (2008); Duellman & Lehr (2009); IUCN (2013) |
| *P. infraguttatus* | 2000 | 2180 | 2090 | 180 | Duellman & Pramuk (1999); Duellman & Lehr (2009) |
| *P. insignitus* | 1530 | 2134 | 1832 | 604 | Stuart *et al*. (2008); Frost (2013); IUCN (2013) |
| *P. inusitatus* | 1300 | 2160 | 1730 | 860 | Guayasamín & Funk (2009); Frost (2013); IUCN (2013) |
| *P. ixalus* | 1300 | 1700 | 1500 | 400 | Lynch (2003a); Stuart *et al*. (2008); Frost (2013); IUCN (2013) |
| *P. jabonensis* | 3100 | 3200 | 3150 | 100 | La Marca (2007) |
| *P. jaimei* | 800 | 1580 | 1190 | 780 | Lynch (1992, 1998); IUCN (2013) |
| *P. johannesdei* | 1410 | 1800 | 1605 | 390 | Lynch (1998); Ruiz-Carranza *et al*. (1996); IUCN (2013) |
| *P. jorgevelosai* | 1900 | 2200 | 2050 | 300 | Lynch (1994b); Acosta-Galvis (2000) |
| *P. juanchoi* | 1500 | 2090 | 1795 | 590 | Stuart *et al*. (2008); IUCN (2013) |
| *P. jubatus* | 2550 | 2750 | 2650 | 200 | García & Lynch (2006); Frost (2013); IUCN (2013) |
| *P. kareliae* | 2500 | 3400 | 2950 | 900 | La Marca (2005); Frost (2013); IUCN (2013) |
| *P. katoptroides* | 1000 | 2700 | 1850 | 1700 | Stuart *et al*. (2008); Frost (2013); IUCN (2013) |
| *P. kelephus* | 1900 | 2250 | 2075 | 350 | Lynch (1998); Frost (2013); IUCN (2013) |
| *P. kirklandi* | 2200 | 2200 | 2200 | 0 | Frost (2013) |
| *P. koehleri* | 437 | 735 | 586 | 298 | Padial & De la Riva (2009) |
| *P. labiosus* | 30 | 1600 | 815 | 1570 | Lynch (1998); Acosta-Galvis (2000); Frost (2013) |
| *P. lacrimosus* | 100 | 1100 | 600 | 1000 | Duellman & Lehr (2009); IUCN (2013) |
| *P. lancinii* | 2500 | 3430 | 2965 | 930 | Donoso-Barros (1965); La Marca (2007); IUCN (2013) |
| *P. lanthanites* | 200 | 1650 | 925 | 1450 | Duellman & Lehr (2009); IUCN (2013) |
| *P. lasalleorum* | 3700 | 3850 | 3775 | 150 | Stuart *et al*. (2008); Frost (2013); IUCN (2013) |
| *P. lassoalcalai* | 1827 | 1950 | 1889 | 123 | Barrio-Amorós *et al*. (2010); Frost (2013) |
| *P. laticlavius* | 1200 | 2565 | 1883 | 1365 | Frost (2013); IUCN (2013) |
| *P. latidiscus* | 2 | 950 | 476 | 948 | Lynch (1998); Frost (2013); IUCN (2013) |
| *P. lemur* | 1800 | 2650 | 2225 | 850 | Stuart *et al*. (2008); Frost (2013); IUCN (2013) |
| *P. lentiginosus* | 1700 | 1800 | 1750 | 100 | Rivero (1982b) |
| *P. leptolophus* | 2800 | 3300 | 3050 | 500 | Lynch (1980); Acosta-Galvis (2000); IUCN (2013) |
| *P. leucopus* | 2300 | 2900 | 2600 | 600 | Acosta-Galvis (2000); Guayasamín & Funk (2009); IUCN (2013) |
| *P. leucorrhinus* | 2500 | 2500 | 2500 | 0 | Duellman & Lehr (2009); Frost (2013) |
| *P. librarius* | 220 | 560 | 390 | 340 | Frost (2013); IUCN (2013) |
| *P. lichenoides* | 2000 | 2450 | 2225 | 450 | Stuart *et al*. (2008); Frost (2013); IUCN (2013) |
| *P. lindae* | 1700 | 1700 | 1700 | 0 | Duellman & Lehr (2009); Frost (2013); IUCN (2013) |
| *P. lirellus* | 470 | 1200 | 835 | 730 | Duellman & Lehr (2009); Frost (2013); IUCN (2013) |
| *P. lividus* | 2135 | 2750 | 2443 | 615 | Almendáriz & Orcés (2004); Duellman & Lehr (2009) |
| *P. llojsintuta* | 2000 | 2200 | 2100 | 200 | Frost (2013); IUCN (2013) |
| *P. loujosti* | 2800 | 2800 | 2800 | 0 | Frost (2013) |
| *P. loustes* | 1200 | 1410 | 1305 | 210 | Stuart *et al*. (2008); Frost (2013); IUCN (2013) |
| *P. lucasi* | 2790 | 3000 | 2895 | 210 | Duellman & Chaparro (2008); Frost (2013); IUCN (2013) |
| *P. lucidosignatus* | 2115 | 2115 | 2115 | 0 | Rödder & Schmitz (2009); Frost (2013) |
| *P. luteolateralis* | 1140 | 1960 | 1550 | 820 | Stuart *et al*. (2008); Frost (2013); IUCN (2013) |
| *P. lutitus* | 1750 | 2400 | 2075 | 650 | Jerez *et al*. (2001); IUCN (2013) |
| *P. lymani* | 450 | 3200 | 1825 | 2750 | Venegas (2005); Duellman & Lehr (2009); IUCN (2013) |
| *P. lynchi* | 2460 | 3340 | 2900 | 880 | IUCN (2013) |
| *P. maculosus* | 2560 | 2900 | 2730 | 340 | Acosta-Galvis (2000); Frost (2013); IUCN (2013) |
| *P. mariaelenae* | 3596 | 3596 | 3596 | 0 | Venegas & Duellman (2012); Frost (2013); IUCN (2013) |
| *P. mars* | 1760 | 1790 | 1775 | 30 | Stuart *et al*. (2008); Frost (2013); IUCN (2013) |
| *P. martiae* | 300 | 1300 | 800 | 1000 | Ruiz-Carranza *et al*. (1996); Duellman & Lehr (2009); Frost (2013) |
| *P. mazar* | 2895 | 3415 | 3155 | 520 | Guayasamin & Arteaga (2013) |
| *P. medemi* | 450 | 1800 | 1125 | 1350 | Malambo & Marin (2006); Frost (2013); IUCN (2013) |
| *P. megalops* | 1300 | 2450 | 1875 | 1150 | Stuart *et al*. (2008); Frost (2013); IUCN (2013) |
| *P. melanogaster* | 2800 | 3470 | 3135 | 670 | Duellman & Lehr (2009); Frost (2013); IUCN (2013) |
| *P. melanoproctus* | 1800 | 1800 | 1800 | 0 | Frost (2013); IUCN (2013) |
| *P. mendax* | 1700 | 3325 | 2513 | 1625 | Duellman & Lehr (2009); Frost (2013) |
| *P. meridionalis* | 2290 | 2290 | 2290 | 0 | Duellman & Lehr (2009); Frost (2013); IUCN (2013) |
| *P. merostictus* | 2400 | 2400 | 2400 | 0 | Stuart *et al*. (2008); Frost (2013); IUCN (2013) |
| *P. minimus* | 1250 | 1685 | 1468 | 435 | Terán-Valdez & Guayasamin (2010); Frost (2013) |
| *P. minutulus* | 1970 | 1970 | 1970 | 0 | Duellman & Hedges (2007); Duellman & Lehr (2009) |
| *P. miyatai* | 1740 | 2400 | 2070 | 660 | Stuart *et al*. (2008); Frost (2013); IUCN (2013) |
| *P. mnionaetes* | 3060 | 3800 | 3430 | 740 | Stuart *et al*. (2008); Frost (2013); IUCN (2013) |
| *P. modipeplus* | 2560 | 3700 | 3130 | 1140 | Stuart *et al*. (2008); Frost (2013); IUCN (2013) |
| *P. molybrignus* | 1110 | 2350 | 1730 | 1240 | Stuart *et al*. (2008); Frost (2013); IUCN (2013) |
| *P. mondolfii* | 1120 | 1120 | 1120 | 0 | Frost (2013); IUCN (2013) |
| *P. muricatus* | 800 | 1380 | 1090 | 580 | Ortega-Andrade *et al*. (2010); Frost (2013); IUCN (2013) |
| *P. muscosus* | 1800 | 2500 | 2150 | 700 | Duellman & Lehr (2009); Yánez-Muñoz *et al*. (2012) |
| *P. myersi* | 2900 | 3275 | 3088 | 375 | Acosta-Galvis (2000); Frost (2013); IUCN (2013) |
| *P. myops* | 1500 | 2250 | 1875 | 750 | Lynch (1998) |
| *P. caliginosus* | 1650 | 1650 | 1650 | 0 | Frost (2013); IUCN (2013) |
| *P. nephophilus* | 1080 | 2500 | 1790 | 1420 | Duellman & Lehr (2009); Frost (2013); IUCN (2013) |
| *P. nervicus* | 3100 | 3870 | 3485 | 770 | Acosta-Galvis (2000); Frost (2013); IUCN (2013) |
| *P. nicefori* | 2500 | 4100 | 3300 | 1600 | Frost (2013); IUCN (2013) |
| *P. nigrogriseus* | 1150 | 2835 | 1993 | 1685 | Stuart *et al*. (2008); IUCN (2013) |
| *P. nyctophylax* | 1140 | 2100 | 1620 | 960 | Stuart *et al*. (2008); Frost (2013); IUCN (2013) |
| *P. obmutescens* | 3200 | 3500 | 3350 | 300 | Lynch (1980); Lynch *et al*. (1996); Frost (2013); IUCN (2013) |
| *P. ocellatus* | 1050 | 2600 | 1825 | 1550 | Lynch & Burrowes (1990); Lynch (1998); IUCN (2013) |
| *P. ockendeni* | 300 | 1200 | 750 | 900 | Duellman & Lehr (2009); IUCN (2013) |
| *P. ocreatus* | 3500 | 4150 | 3825 | 650 | Stuart *et al*. (2008); Frost (2013); IUCN (2013) |
| *P. olivaceus* | 350 | 1650 | 1000 | 1300 | Duellman & Lehr (2009); Frost (2013); IUCN (2013) |
| *P. onorei* | 705 | 2115 | 1410 | 1410 | Rödder & Schmitz (2009); Frost (2013) |
| *P. orcesi* | 3160 | 3800 | 3480 | 640 | Almendáriz & Orcés (2004); Frost (2013); IUCN (2013) |
| *P. orestes* | 2720 | 3120 | 2920 | 400 | Stuart *et al*. (2008); Frost (2013); IUCN (2013) |
| *P. ornatissimus* | 400 | 1800 | 1100 | 1400 | Stuart *et al*. (2008); Frost (2013); IUCN (2013) |
| *P. ornatus* | 2400 | 3000 | 2700 | 600 | Duellman & Lehr (2009); Frost (2013); IUCN (2013) |
| *P. orpacobates* | 700 | 2000 | 1350 | 1300 | Lynch (1998); Acosta-Galvis (2000); IUCN (2013) |
| *P. ortizi* | 3264 | 3420 | 3342 | 156 | Guayasamin *et al*. (2004); Frost (2013); IUCN (2013) |
| *P. padrecarlosi* | 1750 | 1950 | 1850 | 200 | Mueses-Cisneros (2006); Frost (2013); IUCN (2013) |
| *P. paisa* | 1800 | 3100 | 2450 | 1300 | Lynch & Ardila-Robayo (1999); Frost (2013); IUCN (2013) |
| *P. palmeri* | 900 | 2400 | 1650 | 1500 | Bolívar-García *et al*. (2011); Frost (2013); IUCN (2013) |
| *P. paramerus* | 2900 | 3330 | 3115 | 430 | Rivero (1982a) |
| *P. pardalinus* | 2640 | 2640 | 2640 | 0 | Duellman & Lehr (2009); Frost (2013); IUCN (2013) |
| *P. parectatus* | 1800 | 2850 | 2325 | 1050 | Lynch & Rueda-Almonacid (1998b); IUCN (2013) |
| *P. parvillus* | 220 | 2000 | 1110 | 1780 | Ruíz-Carranza *et al*. (1996); Acosta-Galvis (2000); IUCN (2013) |
| *P. pastazensis* | 1800 | 1840 | 1820 | 40 | Stuart *et al*. (2008); Frost (2013); IUCN (2013) |
| *P. pataikos* | 1800 | 3470 | 2635 | 1670 | Stuart *et al*. (2008); Duellman & Lehr (2009); Frost (2013) |
| *P. pecki* | 1138 | 1700 | 1419 | 562 | Duellman & Lehr (2009); IUCN (2013) |
| *P. pedimontanus* | 980 | 1700 | 1340 | 720 | Frost (2013); IUCN (2013) |
| *P. penelopus* | 1180 | 1500 | 1340 | 320 | Stuart *et al*. (2008); Frost (2013); IUCN (2013) |
| *P. peraticus* | 2850 | 3460 | 3155 | 610 | Lynch (1980); Acosta-Galvis (2000); IUCN (2013) |
| *P. percnopterus* | 1380 | 2400 | 1890 | 1020 | Duellman & Lehr (2009); Frost (2013); IUCN (2013) |
| *P. percultus* | 2850 | 2850 | 2850 | 0 | Lynch (1979); Stuart *et al*. (2008); Frost (2013) |
| *P. permixtus* | 1900 | 3700 | 2800 | 1800 | Méndez-Narváez *et al*. (2010); Frost (2013); IUCN (2013) |
| *P. peruvianus* | 200 | 2050 | 1125 | 1850 | Duellman & Lehr (2009); Frost (2013); IUCN (2013) |
| *P. petersi* | 1410 | 2750 | 2080 | 1340 | Mueses-Cisneros (2005); Frost (2013); IUCN (2013) |
| *P. petrobardus* | 1500 | 2500 | 2000 | 1000 | Duellman (1991); Duellman & Lehr (2009); Frost (2013) |
| *P. phalaroinguinis* | 1800 | 2600 | 2200 | 800 | Duellman & Lehr (2007, 2009); Frost (2013); IUCN (2013) |
| *P. phalarus* | 2160 | 2400 | 2280 | 240 | Lynch (1998); Acosta-Galvis (2000); Frost (2013); IUCN (2013) |
| *P. pharangobates* | 1180 | 2759 | 1970 | 1579 | Duellman & Lehr (2009); Frost (2013); IUCN (2013) |
| *P. philipi* | 3580 | 3820 | 3700 | 240 | Lynch & Duellman (1995); Frost (2013); IUCN (2013) |
| *P. phoxocephalus* | 1800 | 3100 | 2450 | 1300 | Duellman & Lehr (2007, 2009); Frost (2013); IUCN (2013) |
| *P. phragmipleuron* | 1800 | 1800 | 1800 | 0 | Stuart *et al*. (2008); Frost (2013); IUCN (2013) |
| *P. piceus* | 2400 | 3400 | 2900 | 1000 | Ruíz-Carranza *et al*. (1996); Frost (2013); IUCN (2013) |
| *P. pinguis* | 3000 | 3916 | 3458 | 916 | Duellman & Pramuk (1999); Duellman & Lehr (2009) |
| *P. platychilus* | 1580 | 2600 | 2090 | 1020 | Lynch (1996); Stuart *et al*. (2008); Frost (2013); IUCN (2013) |
| *P. platydactylus* | 930 | 3470 | 2200 | 2540 | Lehr *et al*. (2006); Duellman & Lehr (2009); IUCN (2013) |
| *P. pleurostriatus* | 2316 | 2316 | 2316 | 0 | Frost (2013); IUCN (2013) |
| *P. polemistes* | 2300 | 2320 | 2310 | 20 | Lynch & Ardila-Robayo (2004); Frost (2013); IUCN (2013) |
| *P. polychrus* | 1140 | 1540 | 1340 | 400 | Ruiz-Carranza *et al*. (1997); Frost (2013); IUCN (2013) |
| *P. prolatus* | 1140 | 1700 | 1420 | 560 | Stuart *et al*. (2008); Frost (2013); IUCN (2013) |
| *P. prolixodiscus* | 1600 | 2700 | 2150 | 1100 | Lynch (1978); Barrio-Amorós (2004); IUCN (2013) |
| *P. proserpens* | 1707 | 2622 | 2165 | 915 | Lynch (1979); Duellman & Lehr (2009); Frost (2013) |
| *P. pteridophilus* | 1500 | 2710 | 2105 | 1210 | Lynch & Duellman (1997); Frost (2013); IUCN (2013) |
| *P. ptochus* | 2100 | 2250 | 2175 | 150 | Lynch (1998); Frost (2013); IUCN (2013) |
| *P. pugnax* | 1660 | 3300 | 2480 | 1640 | Mueses-Cisneros (2005); IUCN (2013) |
| *P. pycnodermis* | 2652 | 3384 | 3018 | 732 | Stuart *et al*. (2008); Frost (2013); IUCN (2013) |
| *P. pyrrhomerus* | 2075 | 3000 | 2538 | 925 | Lynch & Duellman (1997); Frost (2013); IUCN (2013) |
| *P. quantus* | 2100 | 2250 | 2175 | 150 | Lynch (1998); Stuart *et al*. (2008); Frost (2013) |
| *P. quaquaversus* | 200 | 1900 | 1050 | 1700 | Duellman & Pramuk (1999); Duellman & Lehr (2009) |
| *P. quicato* | 2600 | 2900 | 2750 | 300 | Ospina-Sarria *et al*. (2011); Frost (2013) |
| *P. quinquagesimus* | 1410 | 2710 | 2060 | 1300 | Lynch & Duellman (1997); Frost (2013); IUCN (2013) |
| *P. racemus* | 3030 | 3570 | 3300 | 540 | Lynch *et al*. (1996); Frost (2013); IUCN (2013) |
| *P. reclusas* | 2800 | 2800 | 2800 | 0 | Lynch (2003b); Frost (2013); IUCN (2013) |
| *P. reichlei* | 0 | 1500 | 750 | 1500 | Duellman & Lehr (2009); Frost (2013); IUCN (2013) |
| *P. renjiforum* | 2000 | 2800 | 2400 | 800 | Lynch (2000); Stuart *et al*. (2008); Frost (2013); IUCN (2013) |
| *P. repens* | 3150 | 3720 | 3435 | 570 | Rojas & Gutiérrez (2008); Frost (2013); IUCN (2013) |
| *P. restrepoi* | 1790 | 2250 | 2020 | 460 | Lynch (1998); Acosta-Galvis (2000); IUCN (2013) |
| *P. rhabdocnemus* | 230 | 2900 | 1565 | 2670 | Duellman & Lehr (2009) |
| *P. rhabdolaemus* | 300 | 2650 | 1475 | 2350 | Duellman & Lehr (2009) |
| *P. rhigophilus* | 2360 | 3100 | 2730 | 740 | La Marca (2007); Frost (2013) |
| *P. rhodoplichus* | 1800 | 3050 | 2425 | 1250 | Duellman & Lehr (2009); Frost (2013); IUCN (2013) |
| *P. rhodostichus* | 1080 | 1800 | 1440 | 720 | Duellman & Lehr (2009); Yáñez-Muñoz *et al*. (2012); IUCN (2013) |
| *P. ridens* | 10 | 1060 | 535 | 1050 | Lynch (1998); Acosta-Galvis (2000); IUCN (2013) |
| *P. rivasi* | 1389 | 1945 | 1667 | 556 | Barrio-Amorós *et al*. (2010); Frost (2013); IUCN (2013) |
| *P. riveti* | 2620 | 3600 | 3110 | 980 | Stuart *et al*. (2008); Frost (2013); IUCN (2013) |
| *P. romanorum* | 2600 | 2900 | 2750 | 300 | Yánez-Muñoz *et al*. (2010); Frost (2013) |
| *P. roseus* | 0 | 900 | 450 | 900 | Stuart *et al*. (2008); Frost (2013); IUCN (2013) |
| *P. rubicundus* | 1080 | 1300 | 1190 | 220 | Stuart *et al*. (2008); Frost (2013); IUCN (2013) |
| *P. ruedai* | 1000 | 1900 | 1450 | 900 | Lynch (1998); Stuart *et al*. (2008); Frost (2013) |
| *P. rufioculis* | 1138 | 2870 | 2004 | 1732 | Duellman & Lehr (2009) |
| *P. rufoviridis* | 1800 | 2237 | 2019 | 437 | Valencia *et al*. (2010, 2011) |
| *P. ruidus* | 2317 | 2317 | 2317 | 0 | Lynch (1979); Frost (2013); IUCN (2013) |
| *P. ruthveni* | 1800 | 3500 | 2650 | 1700 | Rueda-Solano & Vargas-Salinas (2010); Frost (2013) |
| *P. sagittulus* | 1970 | 2479 | 2225 | 509 | Duellman & Lehr (2009) |
| *P. salaputium* | 1500 | 2400 | 1950 | 900 | Duellman & Lehr (2009); Frost (2013) |
| *P. samaipatae* | 800 | 2000 | 1400 | 1200 | Frost (2013) |
| *P. sanctaemartae* | 1100 | 2600 | 1850 | 1500 | Stuart *et al*. (2008); Frost (2013); IUCN (2013) |
| *P. sanguineus* | 50 | 1500 | 775 | 1450 | Stuart *et al*. (2008); Frost (2013); IUCN (2013) |
| *P. satagius* | 3300 | 3800 | 3550 | 500 | Stuart *et al*. (2008); Frost (2013); IUCN (2013) |
| *P. savagei* | 1000 | 2400 | 1700 | 1400 | Stuart *et al*. (2008); Frost (2013); IUCN (2013) |
| *P. schultei* | 2400 | 2850 | 2625 | 450 | Duellman & Lehr (2009); Frost (2013); IUCN (2013) |
| *P. scitulus* | 2620 | 2620 | 2620 | 0 | Duellman & Lehr (2009); Frost (2013); IUCN (2013) |
| *P. scoloblepharus* | 2620 | 2800 | 2710 | 180 | Stuart *et al*. (2008); Frost (2013); IUCN (2013) |
| *P. scolodiscus* | 1200 | 1780 | 1490 | 580 | Stuart *et al*. (2008); Frost (2013); IUCN (2013) |
| *P. scopaeus* | 3580 | 3600 | 3590 | 20 | Ruíz-Carranza *et al*. (1996) |
| *P. seorsus* | 3350 | 3350 | 3350 | 0 | Duellman & Lehr (2009); IUCN (2013) |
| *P. serendipitus* | 1700 | 1850 | 1775 | 150 | Duellman & Lehr (2009); IUCN (2013) |
| *P. signifer* | 1850 | 1860 | 1855 | 10 | Stuart *et al*. (2008); Frost (2013); IUCN (2013) |
| *P. silverstonei* | 1700 | 2250 | 1975 | 550 | Stuart *et al*. (2008); Frost (2013); IUCN (2013) |
| *P. simonbolivari* | 3200 | 3200 | 3200 | 0 | Wiens & Coloma (1992); Frost (2013); IUCN (2013) |
| *P. simonsii* | 3050 | 3760 | 3405 | 710 | Duellman & Lehr (2009); Frost (2013) |
| *P. simoteriscus* | 3580 | 3680 | 3630 | 100 | Lynch *et al*. (1996); Stuart *et al*. (2008); IUCN (2013) |
| *P. simoterus* | 2700 | 4350 | 3525 | 1650 | Stuart *et al*. (2008); Frost (2013); IUCN (2013) |
| *P. siopelus* | 1700 | 2020 | 1860 | 320 | Lynch & Burrowes (1990); IUCN (2013) |
| *P. sirnigeli* | 2800 | 3050 | 2925 | 250 | Yánez-Muñoz *et al*. (2010); Frost (2013) |
| *P. skydmainos* | 0 | 950 | 475 | 950 | Duellman & Lehr (2009); Cisneros-Heredia *et al*. (2009) |
| *P. sobetes* | 1700 | 2050 | 1875 | 350 | Stuart *et al*. (2008); IUCN (2013) |
| *P. spectabilis* | 3300 | 3300 | 3300 | 0 | Duellman & Lehr (2009); Frost (2013) |
| *P. spilogaster* | 2200 | 2400 | 2300 | 200 | Stuart *et al*. (2008); Frost (2013); IUCN (2013) |
| *P. spinosus* | 1707 | 2835 | 2271 | 1128 | Stuart *et al*. (2008); Frost (2013); IUCN (2013) |
| *P. sternothylax* | 1500 | 4538 | 3019 | 3038 | Duellman & Lehr (2009); Yánez-Muñoz *et al*. (2012) |
| *P. stictoboubonus* | 3000 | 3130 | 3065 | 130 | Duellman & Lehr (2009); IUCN (2013) |
| *P. stictogaster* | 1470 | 2790 | 2130 | 1320 | Duellman & Lehr (2009); Frost (2013) |
| *P. stipa* | 3596 | 3596 | 3596 | 0 | Venegas & Duellman (2012); Frost (2013); IUCN (2013) |
| *P. subsigillatus* | 100 | 930 | 515 | 830 | Lynch & Duellman (1997) |
| *P. suetus* | 1800 | 2800 | 2300 | 1000 | Stuart *et al*. (2008); Frost (2013); IUCN (2013) |
| *P. sulculus* | 1700 | 2020 | 1860 | 320 | Stuart *et al*. (2008); Frost (2013); IUCN (2013) |
| *P. supernatis* | 2280 | 3500 | 2890 | 1220 | Lynch (1979); Frost (2013); IUCN (2013) |
| *P. surdus* | 1550 | 3190 | 2370 | 1640 | Stuart *et al*. (2008); Frost (2013); IUCN (2013) |
| *P. susaguae* | 2530 | 2900 | 2715 | 370 | Stuart *et al*. (2008); Frost (2013); IUCN (2013) |
| *P. taciturnus* | 2400 | 2670 | 2535 | 270 | Lynch & Suárez-Mayorga (2003); IUCN (2013) |
| *P. taeniatus* | 0 | 1400 | 700 | 1400 | IUCN (2013) |
| *P. tamsitti* | 1350 | 2040 | 1695 | 690 | Stuart *et al*. (2008); Frost (2013); IUCN (2013) |
| *P. tanyrhynchus* | 2050 | 2050 | 2050 | 0 | Lehr (2007); Duellman & Lehr (2009); IUCN (2013) |
| *P. tayrona* | 1300 | 2700 | 2000 | 1400 | Stuart *et al*. (2008); Frost (2013); IUCN (2013) |
| *P. telefericus* | 3400 | 3500 | 3450 | 100 | La Marca (2005); IUCN (2013) |
| *P. tenebrionis* | 220 | 830 | 525 | 610 | Lynch & Duellman (1997); Frost (2013); IUCN (2013) |
| *P. thectopternus* | 1580 | 2520 | 2050 | 940 | Lynch (1998) |
| *P. thyellus* | 2900 | 3800 | 3350 | 900 | La Marca (2007); Frost (2013) |
| *P. thymalopsoides* | 2460 | 2480 | 2470 | 20 | Lynch & Duellman (1997); Frost (2013); IUCN (2013) |
| *P. thymelensis* | 3220 | 4150 | 3685 | 930 | Mueses-Cisneros (2005); IUCN (2013) |
| *P. torrenticola* | 1800 | 2400 | 2100 | 600 | Stuart *et al*. (2008); Frost (2013); IUCN (2013) |
| *P. trachyblepharis* | 320 | 1250 | 785 | 930 | Frost (2013); IUCN (2013) |
| *P. tribulosus* | 1900 | 2400 | 2150 | 500 | Stuart *et al*. (2008); Frost (2013); IUCN (2013) |
| *P. truebae* | 2870 | 3190 | 3030 | 320 | Stuart *et al*. (2008); Frost (2013); IUCN (2013) |
| *P. tubernasus* | 1000 | 2300 | 1650 | 1300 | Ruíz-Carranza *et al*. (1996); Frost (2013); IUCN (2013) |
| *P. tungurahua* | 2500 | 2750 | 2625 | 250 | Reyes-Puig *et al*. (2011); Frost (2013) |
| *P. turik* | 1700 | 1700 | 1700 | 0 | Barrio-Amorós *et al*. (2007); Frost (2013) |
| *P. uisae* | 2700 | 2700 | 2700 | 0 | Frost (2013); IUCN (2013) |
| *P. unistrigatus* | 2200 | 3400 | 2800 | 1200 | Lynch & Duellman (1997); Mueses-Cisneros (2005); IUCN (2013) |
| *P. uranobates* | 2800 | 3480 | 3140 | 680 | Lynch (1991); Ruíz-Carranza *et al*. (1996) |
| *P. vanadise* | 1800 | 2600 | 2200 | 800 | Frost (2013); IUCN (2013) |
| *P. variabilis* | 100 | 600 | 350 | 500 | Duellman & Lehr (2009); Frost (2013) |
| *P. veletis* | 1800 | 2450 | 2125 | 650 | Stuart *et al*. (2008); Frost (2013); IUCN (2013) |
| *P. ventriguttatus* | 1700 | 1800 | 1750 | 100 | Lehr & Köhler (2007); IUCN (2013) |
| *P. ventrimarmoratus* | 0 | 1740 | 870 | 1740 | Padial *et al*. (2004); Duellman & Lehr (2009); IUCN (2013) |
| *P. verecundus* | 900 | 2020 | 1460 | 1120 | Stuart *et al*. (2008); Frost (2013); IUCN (2013) |
| *P. versicolor* | 665 | 3100 | 1883 | 2435 | Stuart *et al*. (2008); Frost (2013); IUCN (2013) |
| *P. vertebralis* | 1800 | 3000 | 2400 | 1200 | Lynch & Duellman (1997); Frost (2013); IUCN (2013) |
| *P. vicarius* | 2900 | 3275 | 3088 | 375 | Stuart *et al*. (2008); Frost (2013); IUCN (2013) |
| *P. vidua* | 2710 | 3100 | 2905 | 390 | Stuart *et al*. (2008); Frost (2013); IUCN (2013) |
| *P. viejas* | 800 | 1880 | 1340 | 1080 | Frost (2013); IUCN (2013) |
| *P. vilcabambae* | 2050 | 2050 | 2050 | 0 | Lehr (2007); Frost (2013); IUCN (2013) |
| *P. viridicans* | 1700 | 2680 | 2190 | 980 | Stuart *et al*. (2008); Frost (2013); IUCN (2013) |
| *P. viridis* | 1480 | 1940 | 1710 | 460 | Stuart *et al*. (2008); Frost (2013); IUCN (2013) |
| *P. w-nigrum* | 800 | 3300 | 2050 | 2500 | Duellman & Lehr (2009); Frost (2013); IUCN (2013) |
| *P. wagteri* | 2800 | 3000 | 2900 | 200 | Venegas (2007); Duellman & Lehr (2009); IUCN (2013) |
| *P. walkeri* | 100 | 1270 | 685 | 1170 | Frost (2013); IUCN (2013) |
| *P. wiensi* | 1600 | 1735 | 1668 | 135 | Duellman & Lehr (2009); Frost (2013); IUCN (2013) |
| *P. xeniolum* | 3300 | 3600 | 3450 | 300 | Lynch (2001); Frost (2013); IUCN (2013) |
| *P. xestus* | 4050 | 4050 | 4050 | 0 | Lynch (1995); Frost (2013); IUCN (2013) |
| *P. xylochobates* | 2100 | 2250 | 2175 | 150 | Lynch & Ruiz-Carranza (1996); IUCN (2013) |
| *P. yukpa* | 600 | 1200 | 900 | 600 | Barrio-Amorós *et al*. (2007); Frost (2013) |
| *P. yumbo* | 2400 | 2900 | 2650 | 500 | Yánez-Muñoz *et al*. (2010); Frost (2013) |
| *P. yustizi* | 600 | 1600 | 1100 | 1000 | Frost (2013); IUCN (2013) |
| *P. zoilae* | 2060 | 2550 | 2305 | 490 | Mueses-Cisneros (2007); Frost (2013); IUCN (2013) |
| *P. zophus* | 2030 | 2800 | 2415 | 770 | Lynch & Ardila-Robayo (1999); IUCN (2013) |

**References**

Acosta-Galvis, A.R. (2000) Ranas, salamandras y caecilias (Tetrapoda: Amphibia) de Colombia. *Biota Colombiana*, **1**, 289-319.

Almendáriz, A. & Orcés, G. (2004) Distribución de algunas especies de la herpetofauna de los pisos altoandino, temperado y subtropical. *Politécnica Quito*, **25**, 97-150.

Arteaga-Navarro, A.F. & Guayasamin, J.M. (2011) A new frog of the genus *Pristimantis* (Amphibia: Strabomantidae) from the high Andes of southeastern Ecuador, discovered using morphological and molecular data. *Zootaxa*, **2876**, 17-29.

Arroyo, S.B. (2007) New frog (Brachycephalidae: *Eleutherodactylus*) from the western flank of the Cordillera Oriental of Colombia. *Zootaxa*, **1389**, 61-68.

Barrio-Amorós, C.L. (2004) Anfibios de Venezuela, lista sistemática, distribución y referencias, una aproximación. *Revista de ecología latinoamericana*, **3**, 1-48.

Barrio-Amorós, C.L. (2011) A new *Pristimatis* Jiménez de la Espada, 1870 (Anura: Strabomantidae) from the cloud forests in the Venezuelan Andes. *Anartia*, **23**, 17-26.

Barrio-Amorós, C.L., Rojas-Runjaic, F.J.M. & Infante-Rivero, E.E. (2007) Tres nuevos *Pristimantis* (Anura: Strabomantidae) de la sierra de Perijá, estado Zulia, Venezuela. *Revista Española de Herpetología*, **21**, 71-94.

Barrio-Amorós, C.L., Rojas-Runjaic, F.J.M. & Barros, T.R. (2010) Two new *Pristimantis* (Anura: Terrarana: Strabomantidae) from the Sierra de Perijá, Venezuela. *Zootaxa*, **2329**, 1-21.

Barrio-Amorós, C.L., Guayasamin, J.M. & Hedges, S.B. (2012) A new minute Andean *Pristimantis* (Anura: Strabomantidae) from Venezuela. *Phyllomedusa*, **11**, 83-93.

Barrio-Amorós, C.L., Heinicke, M.P. & Hedges, S.B. (2013) A new tuberculated *Pristimantis* (Anura, Terrarana, Strabomantidae) from the Venezuelan Andes, redescription of *Pristimantis* *pleurostriatus*, and variation within *Pristimantis vanadisae*. *Zootaxa*, **3647**, 043-062.

Bolívar-García, W., Giraldo, A. & Méndez-Narváez, J. (2011) Amphibia, Anura, Strabomantidae, *Pristimantis palmeri* Boulenger, 1912: Distribution extension for the Central Cordillera, Colombia. *Check List*, **7**, 9-10.

Bustamante, M.R. & Mendelson J.R. (2008) A new frog species (Strabomantidae: *Pristimantis*) from the high Andes of southeastern Ecuador. *Zootaxa*, **1820**, 49-59.

Camacho-Badani, T., Yánez-Muñoz, M.H. & Ron, S.R. (2012) *Pristimantis citriogaster* Duellman, 1992 (Amphibia: Craugastoridae): First record from Ecuador, altitudinal distribution extension with distribution map and phylogram. *Check List*, **8**, 513-515.

Chaparro, J.C., Motta, A.P., Gutiérrez, R.C. & Padial, J.M. (2012) A new species of *Pristimantis* (Anura: Strabomantidae) from Andean cloud forests of northern Peru. *Zootaxa*, **3192**, 39-48.

Cisneros-Heredia, D.F., Armijos-Ojeda, D. & Valarezo, K. (2009) First country record of *Pristimantis metabates* (Duellman and Pramuk) and distribution extension of *Pristimantis skydmainos* (Flores and Rodríguez) in eastern Ecuador (Amphibia, Anura, Strabomantidae). *Herpetology Notes*, **2**, 185-188.

Donoso-Barros, R. (1965) Nuevos reptiles y anfibios de Venezuela. *Noticiario Mensual. Museo Nacional de Historia Natural*, **102**, 2-3.

Duellman, W.E. (1991) A new species of *Eleutherodactylus* (Anura: Leptodactylidae) from the Cordillera Occidental of Peru. *Herpetologica*, **47**, 6-9.

Duellman, W.E. (1992) *Eleutherodactylus bearsei* new species (Anura: Leptodactylidae) from northeastern Peru. *Occasional Papers of the Museum of Natural History University of Kansas*, **150**, 1-7.

Duellman, W.E. & Chaparro, J.C. (2008) Two distinctive new species of *Pristimantis* (Anura: Strabomantidae) from the Cordillera Oriental with a distributional synopsis of strabomantids in Central Peru. *Zootaxa*, **1918**, 13-25.

Duellman, W.E. & Hedges, S.B. (2007) Three new species of *Pristimantis* (Lissamphibia, Anura) from montane forests of the Cordillera Yanachaga in Central Peru. *Phyllomedusa*, **6**, 119-135.

Duellman, W.E. & Lehr, E. (2007) Frogs of the genus *Eleutherodactylus* (Leptodactylidae) in the Cordillera Occidental in Peru with descriptions of three new species. *Scientific Papers. Natural History Museum, University of Kansas*, **39**, 1-13.

Duellman, W.E. & Lehr, E. (2009) *Terrestrial-Breeding Frogs (Strabomantidae) in Peru*. Natur und Tier Verlag, Münster, Germany.

Duellman, W.E. & Lynch, J.D. (1988) Anuran amphibians from the Cordillera de Cutucu, Ecuador. *Proceedings of the Academy of Natural Sciences of Philadelphia*, **140**, 125-142.

Duellman, W.E. & Pramuk, J.B. (1999) Frogs of the genus Eleutherodactylus (Anura: Leptodactylidae) in the Andes of Northern Peru. *Scientific Papers. Natural History Museum, University of Kansas,* **13**, 1-78.

Elmer, K.R. & Cannatella, D.C. (2008) Three new species of leaflitter frogs from the upper Amazon forests: cryptic diversity within *Pristimantis ockendeni* (Anura: Strabomantidae) in Ecuador. *Zootaxa*, **1784**, 11-38.

Frenkel, C., Guayasamín, J.M., Yanez-Muñoz, M.H. & Ron, S. (2011) *Pristimantis ganonotus*. AmphibiaWeb Ecuador. Available at: <http://zoologia.puce.edu.ec/vertebrados/anfibios/FichaEspecie.aspx?=1411> (accessed 15 December 2013).

Frost, D.R. (2013) Amphibian species of the world: an online reference. Available at: <http://research.amnh.org/herpetology/amphibia/index.html> (accessed December 2013).

Galvis-Peñuela, P.A. & Rueda-Almonacid, J.V. (2004) Rana de lluvia “de ojos rojos y amarillos” *Eleutherodactylus actinolaimus*. *Libro Rojo de los Anfibios de Colombia* (ed. by J.V. Rueda-Almonacid, J.D. Lynch and A. Amézquita), pp. 313-317. Panamericana Formas e Impresos, Bogotá, Colombia.

García, J.C. & Lynch, J.D. (2006) A new species of frog (genus *Eleutherodactylus*) from a cloud forest in western Colombia. *Zootaxa*, **1171**, 39-45.

Guayasamin, J.M. & Arteaga, A.F. (2013) A new species of the *Pristimantis orestes* group (Amphibia: Strabomantidae) from the high Andes of Ecuador, Reserva Mazar. *Zootaxa*, **3616**, 345-356.

Guayasamin, J.M. & Funk, W.C. (2009) The amphibian community at Yanayacu Biological Station, Ecuador, with a comparison of vertical microhabitat use among *Pristimantis* species and the description of a new species of the *Pristimantis myersi* group. *Zootaxa*, **2220**, 41-66.

Guayasamin, J.M., Almeida-Reinoso, D. & Nogales-Sornosa, F. (2004) Two new species of frogs (Leptodactylidae: *Eleutherodactylus*) from the high Andes of northern Ecuador. *Herpetological Monographs*, **18**, 127-141.

IUCN (2013) The IUCN Red List of Threatened Species. Version 2013.1. Available at: <http://www.iucnredlist.org> (accessed December 2013).

Jerez, A., Arroyo, S. & Ramírez-Pinilla, M.P. (2001) *Eleutherodactylus lutitus*. Geographic distribution. *Herpetological Review* **32**, 270.

La Marca, E. (2005) Dos nuevas especies de rana (Amphibia: Leptodactylidae) de páramo del Parque Nacional Sierra Nevada, Venezuela. *Herpetotropicos*, **2**, 47-54.

La Marca, E. (2007) Sinopsis taxonómica de dos géneros nuevos de anfibios (Anura: Leptodactylidae) de los Andes de Venezuela. *Herpetotropicos*, **3**, 67-87.

La Marca, E. & Smith, H.M. (1982) *Eleutherodactylus colostichos*, a new frog species from the Paramo de los Conejos, in the Venezuelan Andes (Anura: Leptodactylidae). *Occasional Papers of the Museum of Zoology, University of Michigan*, **700**, 1-8.

Lehr, E. (2007) New Eleutherodactyline frogs (Leptodactylidae: *Pristimantis*, *Phrynopus*) from Peru. *Bulletin of the Museum of Comparative Zoology*, **159**, 145-178.

Lehr, E. & Coloma, L.A. (2008) A minute new Ecuadorian Andean frog (Anura: Strabomantidae, *Pristimantis*). *Herpetologica*, **64**, 354-367.

Lehr, E. & Köhler, G. (2007) A new species of the *Pristimantis orestes* group (Anura: Leptodactylidae) from the Cordillera Occidental in northern Peru. *Zootaxa*, **1621**, 45-54.

Lehr, E., Lundberg, M., Aguilar, C. & von May, R. (2006) New species of *Eleutherodactylus* (Anura: Leptodactylidae) from the eastern Andes of central Peru with comments on central Peruvian *Eleutherodactylus*. *Herpetological Monographs*, **20**, 105-128.

Lehr, E., Aguilar, C., Siu-Ting, K. & Jordán, J.C. (2007) Three new species of *Pristimantis* (Anura: Leptodactylidae) from the Cordillera de Huancabamba in northern Peru. *Herpetologica*, **63**, 519-536.

Lynch, J.D. (1978) A new Eleutherodactyline frog from the Andes of northern Colombia. *Copeia*, **1978**, 17-21.

Lynch, J.D. (1979) Leptodactylid frogs of the genus *Eleutherodactylus* from the Andes of southern Ecuador. *Miscellaneous Publications of the Museum of Natural History University of Kansas*, **66**, 1-62.

Lynch, J.D. (1980) New species of *Eleutherodactylus* of Colombia (Amphibia: Leptodactylidae) I: Five new species from the paramos of the Cordillera Central. *Caldasia*, **13**, 165-88.

Lynch, J.D. (1981) Two new species of *Eleutherodactylus* from western Colombia (Amphibia: Anura: Leptodactylidae). *Occasional Papers of the Museum of Zoology, University of Michigan*, **697**, 1-12.

Lynch, J.D. (1986) New species of *Eleutherodactylus* of Colombia (Amphibia: Leptodactylidae) II: Four species from the cloud forests of the western cordilleras. *Caldasia*, **15**, 629-647.

Lynch, J.D. (1991) New diminutive *Eleutherodactylus* from the Cordillera Central of Colombia (Amphibia: Leptodactylidae). *Journal of Herpetology*, **25**, 344-352.

Lynch, J.D. (1992) Two new species of *Eleutherodactylus* from southwestern Colombia and the proposal of a new species group (Amphibia: Leptodactylidae). *Journal of Herpetology*, **26**, 53-59.

Lynch, J.D. (1994a) Two new species of the *Eleutherodactylus conspicillatus* group (Amphibia: Leptodactylidae) from the Cordillera Oriental of Colombia. *Revista de la Academia Colombiana de Ciencias Exactas, Físicas y Naturales*, **19**, 187-193.

Lynch, J. D. (1994b) A new species of frog (genus *Eleutherodactylus*: Leptodactylidae) from a cloud forest in Departamento de Santander, Colombia. *Revista de la Academia Colombiana de Ciencias Exactas, Físicas y Naturales*, **19**, 205-208.

Lynch, J.D. (1995) Three new species of *Eleutherodactylus* (Amphibia: Leptodactylidae) from the paramos of the Cordillera Occidental of Colombia. *Journal of Herpetology*, **29**, 513-521.

Lynch, J.D. (1996) New frogs of the genus *Eleutherodactylus* (family Leptodactylidae) from the San Antonio region of the Colombian Cordillera Occidental. *Revista de la Academia Colombiana de Ciencias Exactas, Físicas y Naturales,* **20**, 331-345.

Lynch, J.D. (1998) New species of *Eleutherodactylus* from Cordillera Occidental of western Colombia with a synopsis of the distributions of species in western Colombia. *Revista de la Academia Colombiana de Ciencias Exactas, Físicas y Naturales*, **22**, 117-148.

Lynch, J.D. (2000) A new species of frog genus *Eleutherodactylus* (Leptodactylidae) from the Sabana de Bogotá. *Revista de la Academia Colombiana de Ciencias Exactas, Físicas y Naturales*, **24**, 435-439.

Lynch, J.D. (2001) A small amphibian fauna from a previously unexplored paramo of the Cordillera Occidental in western Colombia. *Journal of Herpetology*, **35**, 226-231.

Lynch, J.D. (2003a) New species of frogs (*Eleutherodactylus*: Leptodactylidae) from the Cordillera Oriental of Norte de Santander and Santander, Colombia. *Revista de la Academia Colombiana de Ciencias Exactas, Físicas y Naturales*, **27**, 449-460.

Lynch, J.D. (2003b) Two new frogs (*Eleutherodactylus*) from the Serranía de Perijá, Colombia. *Revista de la Academia Colombiana de Ciencias Exactas, Físicas y Naturales*, **27**, 613-617.

Lynch, J.D. & Ardila-Robayo, M.C. (1999) The *Eleutherodactylus* of the *taeniatus* complex in western Colombia: taxonomy and distribution. *Revista de la Academia Colombiana de Ciencias Exactas, Físicas y Naturales*, **23**, 615-624.

Lynch, J.D. & Ardila-Robayo, M.C. (2004) A new Colombian frog of the genus *Eleutherodactylus* from the northern Cordillera Occidental. *Revista de la Academia Colombiana de Ciencias Exactas, Físicas y Naturales*, **28**, 403-408.

Lynch, J.D. & Burrowes, P. (1990) The frogs of the genus *Eleutherodactylus* (family Leptodactylidae) at the La Planada Reserve in southwestern Colombia with descriptions of eight new species. *Occasional Papers of the Museum of Natural History University of Kansas*, **136**, 1-31.

Lynch, J.D. & Duellman, W.E. (1980) The *Eleutherodactylus* of the Amazonian slopes of the Ecuadorian Andes (Anura: Leptodactylidae). *Miscellaneous Publications of the Museum of Natural History University of Kansas*, **69**, 1-86.

Lynch, J.D. & Duellman, W.E. (1995) A new fat little frog (Leptodactylidae: *Eleutherodactylus*) from lofty Andean grasslands of southern Ecuador. *Occasional Papers of the Museum of Natural History University of Kansas*, **173**, 1-7.

Lynch, J.D. & Duellman, W.E. (1997) Frogs of the genus *Eleutherodactylus* in western Ecuador. Systematics, ecology, and biogeography. *Special Publication of the Museum of Natural History University of Kansas*, **23**, 1-236.

Lynch, J.D. & Rueda-Almonacid, J.V. (1998a) Additional new species of frogs (genus *Eleutherodactylus*) from cloud forests of eastern Departamento de Caldas, Colombia. *Revista de la Academia Colombiana de Ciencias Exactas, Físicas y Naturales*, **22**, 287-298.

Lynch, J.D. & Rueda-Almonacid, J.V. (1998b) New frogs of the genus *Eleutherodactylus* from the eastern flank of the northern Cordillera Central of Colombia. *Revista de la Academia Colombiana de Ciencias Exactas, Físicas y Naturales*, **22**, 561-570.

Lynch, J.D. & Rueda-Almonacid, J.V. (1999) New species of frogs from low and moderate elevations from the Caldas transect of the eastern flank of the Cordillera Central. *Revista de la Academia Colombiana de Ciencias Exactas, Físicas y Naturales*, **23**, 307-314.

Lynch, J.D. & Ruiz-Carranza, P.M. (1996) New sister-species of *Eleutherodactylus* from the Cordillera Occidental of southwestern Colombia (Amphibia: Salientia: Leptodactylidae). *Revista de la Academia Colombiana de Ciencias Exactas, Físicas y Naturales*, **20**, 347-363.

Lynch, J.D. & Suárez-Mayorga, A.M. (2000) A new frog (*Eleutherodactylus*: Leptodactylidae) from the southern part of the Cordillera Oriental of Colombia. *Revista de la Academia Colombiana de Ciencias Exactas, Físicas y Naturales*, **24**, 289-293.

Lynch, J.D. & Suárez-Mayorga, A.M. (2003) Two additional new species of *Eleutherodactylus* (Leptodactylidae) from southwestern Colombia. *Revista de la Academia Colombiana de Ciencias Exactas, Físicas y Naturales*, **27**, 607-612.

Lynch, J.D., Ruiz-Carranza, P.M. & Ardila-Robayo, M.C. (1996) Three new species of *Eleutherodactylus* (Amphibia: Leptodactylidae) from high elevations of the Cordillera Central of Colombia. *Caldasia*, **18**, 329-342.

Malambo, C. & Marin, A. (2006) Geographic distribution: *Eleutherodactylus medemi*. *Herpetological Review*, **37**, 487.

Méndez-Narváez, J., Bolívar-García, W. & Castro-Herrera, F. (2010) Amphibia, Anura, Strabomantidae, *Pristimantis permixtus* Lynch, Ruiz-Carranza, and Ardila-Robayo, 1994: Distribution extension, Valle del Cauca, Colombia. *Check List*, **6**, 499-500.

MHUA (2013) Museo de Herpetología Universidad de Antioquía. Available at: <http://www.herpetologicodeantioquia.org> (accessed 10 November 2013).

Mueses-Cisneros, J.J. (2005) Fauna anfibia del Valle de Sibundoy, Putumayo, Colombia. *Caldasia*, **27**, 229-242.

Mueses-Cisneros, J.J. (2006) A new species of *Eleutherodactylus* (Amphibia: Anura: Brachycephalidae) from the western flank of the Cordillera Oriental of Colombia. *Zootaxa*, 1271, 29-35.

Mueses-Cisneros, J.J. (2007) Two new species of the genus *Eleutherodactylus* (Anura: Brachycephalidae) from Valle de Sibundoy, Putumayo, Colombia. *Zootaxa*, **1498**, 35-43.

Ortega-Andrade, H.M., Bermingham, J., Aulestia, C. & Paucar. C. (2010) Herpetofauna of the Bilsa Biological Station, province of Esmeraldas, Ecuador. *Check List*, **6**, 119-154.

Ospina-Sarria, J.J., Méndez-Narváez, J., Burbano-Yandi, C.E. & Bolívar-García, W. (2011) A new species of *Pristimantis* (Amphibia: Craugastoridae) with cranial crests from the Colombian Andes. *Zootaxa*, **3111**, 37-48.

Padial, J.M. & De la Riva, I. (2009) Integrative taxonomy reveals cryptic Amazonian species of *Pristimantis* (Anura: Strabomantidae). *Zoological Journal of the Linnean Society*, **155**, 97-122.

Padial, J.M., Gonzáles-Álvarez, L., Reichle, S., Aguayo-Vedia, C.R. & De la Riva, I. (2004) First records of five species of the genus *Eleutherodactylus* Dumeril and Bibron, 1841 (Anura, Leptodactylidae) for Bolivia. *Graellsia*, **60**, 167-174.

Reyes-Puig, J.P. & Yánez-Muñoz, M.H. (2012) Una nueva especie de *Pristimantis* (Anura: Craugastoridae) del corredor ecológico Llangantes-Sangay, Andes de Ecuador. *Papéis Avulsos de Zoologia (São Paulo),* **52**, 81-91.

Reyes-Puig, J.P., Yánez-Muñoz, M.H., Cisneros-Heredia, D.F. & Ramírez, S. (2011) Una nueva especie de rana *Pristimantis* (Terrarana: Strabomantidae) de los bosques nublados de la cuenca alta del río Pastaza, Ecuador. *Avances en Ciencias e Ingeníerias*, **2**, 78-82.

Rivero, J.A. (1982a) Los *Eleutherodactylus* (Amphibia, Salientia) de los Andes Venezolanos. I. Especies del páramo. *Memoria de la Sociedad de Ciencias Naturales La Salle*, **42**, 17-56.

Rivero, J.A. (1982b) Los *Eleutherodactylus* (Amphibia, Salientia) de los Andes Venezolanos. II, Especies sub-parameras. *Memoria de la Sociedad de Ciencias Naturales La Salle*, **42**, 57-132.

Rödder, D. & Schmitz, A. (2009) Two new *Pristimantis* (Anura, Strabomantidae) belonging to the *myersi* group from the Andean slopes of Ecuador. *Revue Suisse de Zoologie*, **116**, 275-288.

Rojas, M.A. & Gutiérrez, P.D.A. (2008) *Pristimantis repens* (Lynch, 1984), a frog not restricted to the Paramo. *Herpetozoa*, **21**, 85-86.

Rojas, M.A., Gutiérrez, P.D.A. & Cortés-Bedoya, S. (2013) *Pristimantis achatinus* (Boulenger, 1898). *Catálogo de Anfibios y Reptiles de Colombia*, **1**, 35-44.

Rueda-Almonacid, J.V. & Lynch, J.D. (1983) Una nueva especie de *Eleutherodactylus* (Amphibia: Leptodactylidae) para la Cordillera Oriental de Colombia. *Lozania*, *42*, 1-6.

Rueda-Solano, L.A. & Vargas-Salinas, F. (2010) *Pristimantis cristinae* and *Pristimantis ruthveni* Lynch and Ruiz-Carranza, 1985 increase of altitudinal distribution and paramo habitat use in the Sierra Nevada de Santa Marta, Colombia. *Herpetozoa*, **23**, 88-90.

Ruiz-Carranza, P.M., Ardila-Robayo, M.C. & Lynch, J.D. (1996) Lista actualizada de la fauna Amphibia de Colombia. *Revista de la Academia Colombiana de Ciencias Exactas, Físicas y Naturales*, **20**, 365-415.

Ruiz-Carranza, P.M., Lynch, J.D. & Ardila-Robayo, M.C. (1997) Seis nuevas especies de *Eleutherodactylus* Duméril, Bibron, 1841 (Amphibia: Leptodactylidae) del Norte de la Cordillera Occidental de Colombia. *Revista de la Academia Colombiana de Ciencias Exactas, Físicas y Naturales*, **21**, 155-174.

Stuart, S.N., Hoffmann, M., Chanson, J., Cox, N., Berridge, R., Ramani, P. & Young, B. (2008) *Threatened Amphibians of the World*. Lynx Editions, Barcelona, Spain; IUCN, Gland, Switzerland; and Conservation International, Arlington, Virginia, USA.

Terán-Valdez, A. & Guayasamin, J.M. (2010) The smallest terrestrial vertebrate of Ecuador: A new frog of the genus *Pristimantis* (Amphibia: Strabomantidae) from the Cordillera del Cóndor. *Zootaxa*, **2447**, 53-68.

Valencia, J.H., Yánez-Muñoz, M.H., Betancourt-Yépez, R., Terán-Valdez, A. & Guayasamin, J.M. (2010) Una llamativa nueva especie de *Pristimantis* (Anura: Terrarana: Strabomantidae) de las estribaciones noroccidentales de los Andes de Ecuador. *Avances en Ciencias e Ingeníerias*, **2**, B41-B45.

Valencia, J.H., Yánez-Muñoz, M.H., Betancourt-Yépez, R., Terán-Valdez, A. & Guayasamin, J.M (2011) Reemplazo del nombre *Pristimantis viridis* Valencia, Yánez-Muñoz, Betancourt-Yépez y Guayasamin, 2010. *Avances en Ciencias e Ingeníerias*, **3**, B1.

Venegas, P.J. (2005) Herpetofauna del bosque seco ecuatorial de Perú: Taxonomía, ecología y biogeografía. *Zonas Áridas*, **9**, 9-26.

Venegas, P.J. (2007) A new species of *Eleutherodactylus* (Anura: Leptodactylidae) from the Cordillera Central in northern Peru. *Journal of Herpetology*, **41**, 394-400.

Venegas, P.J. & Duellman, W.E. (2012) Two syntopic new species of the *Pristimantis orestes* Group (Anura: Strabomantidae) from northwestern Peru. *Zootaxa*, **3249**, 47-59.

Wiens, J.J. & Coloma, L.A. (1992) A new species of the *Eleutherodactylus myersi* (Anura: Leptodactylidae) assembly from Ecuador. *Journal of Herpetology*, **26**, 196-207.

Yánez-Muñoz, M.H., Meza-Ramos, P., Cisneros-Heredia, D.F. & Reyes-Puig, J.P. (2010) Descripción de tres nuevas especies de ranas del género *Pristimantis* (Anura: Terrarana: Strabomantidae) de los bosques nublados del Distrito Metropolitano de Quito, Ecuador. *Avances en Ciencias e Ingeníerias*, **3**, B16-B27.

Yánez-Muñoz, M.H., Toral-Contreras, E., Meza-Ramos, P., Reyes-Puig, J.P., Bejarano-Muñoz, P., Mueses-Cisneros, J.J. & Paucar, C. (2012) New country records for five species of *Pristimantis* Jiménez de la Espada, 1870 from Ecuador. *Check List*, **8**, 286-290.

**Table S2.** Species-area effect using non-transformed variables (linear effect), log-transformed variables (curvilinear effect), and log-transformed area (semi-log effect). Significant area effects are marked with asterisks (*). The models with the lowest AICc are shown in boldface type. Mountain ranges are sorted by domain and latitude from north to south.

| **Geographic region** | **Linear effect** | | **Curvilinear effect** | | **Semi-log effect** | |
| --- | --- | --- | --- | --- | --- | --- |
|  | *r^2^* | *p-value* | *r^2^* | *p-value* | *r^2^* | *p-value* |
| **Northern Andes domain** |  |  |  |  |  |  |
| Meridan Andes, Venezuela | -0.140 | 0.903 | **0.396** | **0.041*** | 0.081 | 0.233 |
| Sierra Nevada de Santa Marta, Colombia | -0.131 | 0.796 | **-0.068** | **0.505** | -0.067 | 0.503 |
| Eastern Cordillera, Colombia | -0.108 | 0.654 | **0.590** | **0.009*** | 0.279 | 0.083 |
| Central Cordillera, Colombia | -0.050 | 0.457 | **0.624** | **0.007*** | 0.312 | 0.069 |
| Western Cordillera, Colombia | 0.412 | 0.037* | **0.904** | **0.052** | 0.654 | 0.010* |
| Eastern Cordillera, Ecuador | 0.063 | 0.255 | **0.719** | **0.002*** | 0.391 | 0.042* |
| Western Cordillera, Ecuador | 0.251 | 0.097 | **0.740** | **0.002*** | 0.433 | 0.032* |
| **Central Andes domain** |  |  |  |  |  |  |
| Eastern Cordillera, Peru | -0.105 | 0.638 | **-0.141** | **0.914** | -0.136 | 0.841 |
| Central Cordillera, Peru | 0.064 | 0.253 | **0.360** | **0.052** | 0.313 | 0.068 |
| Western Cordillera, Peru | 0.160 | 0.156 | **0.121** | **0.190** | 0.175 | 0.145 |
| Eastern Cordillera, Bolivia | 0.476 | 0.024* | **0.296** | **0.075** | 0.324 | 0.064 |

**Table S3.** Fitting of the spatial constraint effects model (MDE) for empirical and corrected biodiversity curves using linear and quadratic regressions statistics for each main mountain ranges studied here. Significant MDE are marked with asterisks (*). The models with the lowest AICc are shown in boldface type. Mountain ranges are sorted by domain and latitude from north to south.

| **Geographic region** | **Empirical curves** | | | | **Corrected curves** | | | |
| --- | --- | --- | --- | --- | --- | --- | --- | --- |
|  | Linear | | Quadratic | | Linear | | Quadratic | |
|  | *r^2^* | *p-value* | *r^2^* | *p-value* | *r^2^* | *p-value* | *r^2^* | *p-value* |
| **Northern Andes domain** |  |  |  |  |  |  |  |  |
| Meridan Andes, Venezuela | 0.631 | 0.020 | **0.728** | **0.033** | 0.326 | 0.105 | **0.606** | **0.069** |
| Sierra Nevada de Santa Marta, Colombia | **0.862** | **0.004*** | 0.818 | 0.036 | - | - | - | - |
| Eastern Cordillera, Colombia | 0.822 | 0.001* | **0.970** | **<0.001*** | **0.643** | **0.010** | 0.589 | 0.047 |
| Central Cordillera, Colombia | 0.851 | <0.001* | **0.881** | **0.002*** | 0.865 | <0.001* | **0.901** | **0.001*** |
| Western Cordillera, Colombia | **0.168** | **0.172** | 0.002 | 0.429 | - | - | - | - |
| Eastern Cordillera, Ecuador | **0.702** | **0.006** | 0.647 | 0.032 | 0.793 | 0.002* | **0.830** | **0.006** |
| Western Cordillera, Ecuador | **0.219** | **0.115** | 0.104 | 0.303 | **0.182** | **0.140** | 0.046 | 0.366 |
| **Central Andes domain** |  |  |  |  |  |  |  |  |
| Eastern Cordillera, Peru | **-0.051** | **0.434** | -0.398 | 0.768 | - | - | - | - |
| Central Cordillera, Peru | 0.935 | 0.001* | **0.964** | **0.003*** | - | - | - | - |
| Western Cordillera, Peru | 0.813 | <0.001* | **0.922** | **<0.001*** | - | - | - | - |
| Eastern Cordillera, Bolivia | -0.231 | 0.816 | **0.648** | **0.097** | - | - | - | - |

**Table S4.** Effect of spatial topographic heterogeneity effect on species richness using non-transformed variables (linear effect), log-transformed variables (curvilinear effect), and log-transformed area (semi-log effect). Significant topographic heterogeneity effects are indicated with asterisk (*). The models with the lowest AICc are shown in boldface type. Mountain ranges are sorted by domain and latitude from north to south.

| **Geographic region** | **Linear effect** | | **Curvilinear effect** | | **Semi-log effect** | |
| --- | --- | --- | --- | --- | --- | --- |
|  | *r^2^* | *p-value* | *r^2^* | *p-value* | *r^2^* | *p-value* |
| **Northern Andes domain** |  |  |  |  |  |  |
| Meridan Andes, Venezuela | 0.616 | 0.007* | **0.769** | **0.001*** | 0.664 | 0.005* |
| Sierra Nevada de Santa Marta, Colombia | 0.133 | 0.191 | **0.295** | **0.075** | 0.198 | 0.128 |
| Eastern Cordillera, Colombia | 0.892 | <0.001* | **0.847** | **<0.001*** | 0.833 | <0.001* |
| Central Cordillera, Colombia | 0.502 | 0.020* | **0.354** | **0.053** | 0.439 | 0.031* |
| Western Cordillera, Colombia | 0.569 | 0.011* | **0.767** | **0.001*** | 0.492 | 0.021* |
| Eastern Cordillera, Ecuador | -0.008 | 0.366 | **-0.103** | **0.632** | -0.047 | 0.450 |
| Western Cordillera, Ecuador | -0.107 | 0.649 | **-0.103** | **0.630** | -0.112 | 0.674 |
| **Central Andes domain** |  |  |  |  |  |  |
| Eastern Cordillera, Peru | 0.647 | 0.005* | **0.445** | **0.030*** | 0.483 | 0.023* |
| Central Cordillera, Peru | -0.134 | 0.824 | **-0.14** | **0.899** | -0.142 | 0.952 |
| Western Cordillera, Peru | -0.108 | 0.652 | **-0.079** | **0.541** | -0.045 | 0.445 |
| Eastern Cordillera, Bolivia | 0.573 | 0.011* | **0.299** | **0.074** | 0.570 | 0.011* |

**Appendix S2. Supporting figures**

**Figure S1.** Area profiles (open squares and dotted lines) and diversity pattern (solid circles and solid lines) along elevational gradients on main mountain ranges of Tropical Andes. (A) Meridan Andes, Venezuela. (B) Sierra Nevada de Santa Marta, Colombia. (C) Eastern Cordillera, Colombia. (D) Central Cordillera, Colombia. (E) Western Cordillera, Colombia. (F) Eastern Cordillera, Ecuador. (G) Western Cordillera, Ecuador. (H) Eastern Cordillera, Peru. (I) Central Cordillera, Peru. (J) Western Cordillera, Peru. (K) Eastern Cordillera, Bolivia.

**
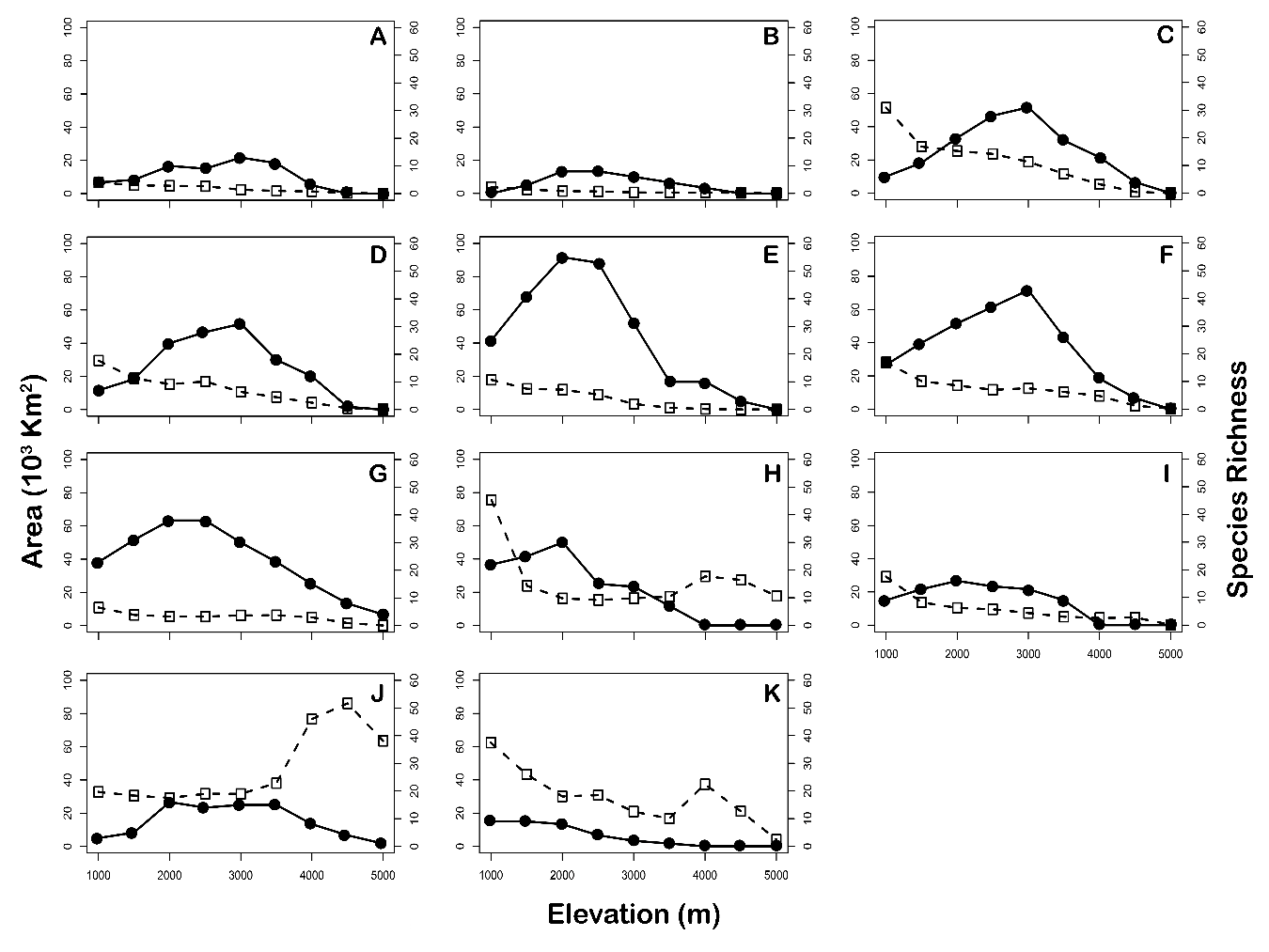
**

**Figure S2.** Comparisons among curvilinear area correction method (solid circles and solid lines) and empirical diversity patterns (open squares and dotted lines) for each main mountain ranges where significant curvilinear species-area effects were detected. (A) Meridan Andes, Venezuela. (B) Eastern Cordillera, Colombia. (C) Central Cordillera, Colombia. (D) Eastern Cordillera, Ecuador. (E) Western Cordillera, Ecuador.**
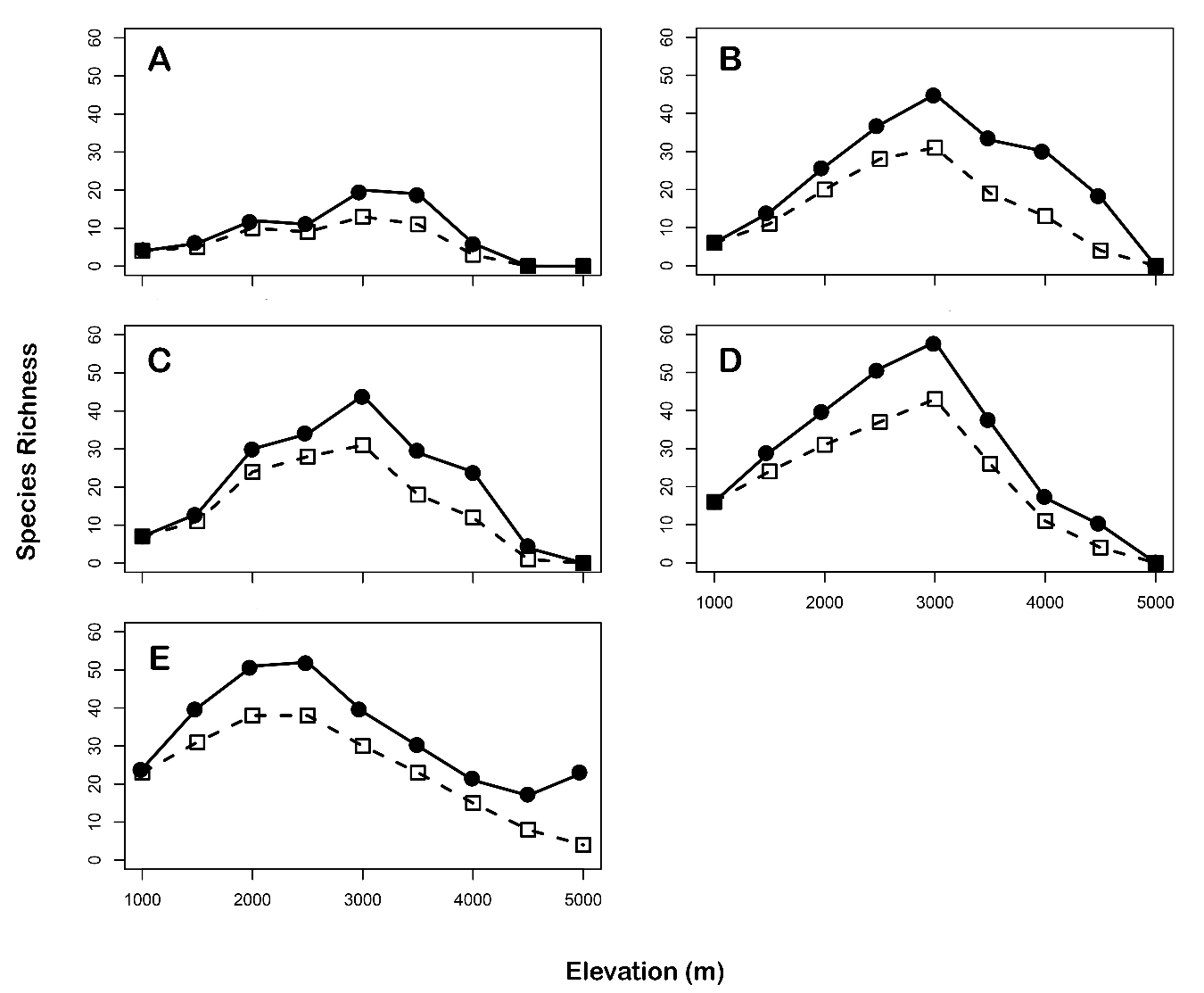
**
